# Inflammatory Foreign Body Response Induced by Neuro-Implants in Rat Cortices Depleted of Resident Microglia by a CSF1R Inhibitor and Its Implications

**DOI:** 10.3389/fnins.2021.646914

**Published:** 2021-03-26

**Authors:** Aviv Sharon, Maciej M. Jankowski, Nava Shmoel, Hadas Erez, Micha E. Spira

**Affiliations:** ^1^Department of Neurobiology, The Alexander Silberman Institute of Life Science, The Hebrew University of Jerusalem, Jerusalem, Israel; ^2^The Charles E. Smith Family and Prof. Joel Elkes Laboratory for Collaborative Research in Psychobiology, The Hebrew University of Jerusalem, Jerusalem, Israel; ^3^Edmond and Lily Safra Center for Brain Sciences, The Hebrew University of Jerusalem, Jerusalem, Israel; ^4^The Harvey M. Kruger Family Center for Nanoscience, The Hebrew University of Jerusalem, Jerusalem, Israel

**Keywords:** bioelectronics, neuro-implant, microglia, PLX5622, CSF1R, foreign body response, field potential recordings, neural interface

## Abstract

Inflammatory encapsulation of implanted cortical-neuro-probes [the foreign body response (FBR)] severely limits their use in basic brain research and in clinical applications. A better understanding of the inflammatory FBR is needed to effectively mitigate these critical limitations. Combining the use of the brain permeant colony stimulating factor 1 receptor inhibitor PLX5622 and a perforated polyimide-based multielectrode array platform (PPMP) that can be sectioned along with the surrounding tissue, we examined the contribution of microglia to the formation of inflammatory FBR. To that end, we imaged the inflammatory processes induced by PPMP implantations after eliminating 89–94% of the cortical microglia by PLX5622 treatment. The observations showed that: (I) inflammatory encapsulation of implanted PPMPs proceeds by astrocytes in microglia-free cortices. The activated astrocytes adhered to the PPMP’s surfaces. This suggests that the roles of microglia in the FBR might be redundant. (II) PPMP implantation into control or continuously PLX5622-treated rats triggered a localized surge of microglia mitosis. The daughter cells that formed a “cloud” of short-lived (*T*_1__/__2_ ≤ 14 days) microglia around and in contact with the implant surfaces were PLX5622 insensitive. (III) Neuron degeneration by PPMP implantation and the ensuing recovery in time, space, and density progressed in a similar manner in the cortices following 89–94% depletion of microglia. This implies that microglia do not serve a protective role with respect to the neurons. (IV) Although the overall cell composition and dimensions of the encapsulating scar in PLX5622-treated rats differed from the controls, the recorded field potential (FP) qualities and yield were undistinguishable. This is accounted for by assuming that the FP amplitudes in the control and PLX5622-treated rats were related to the seal resistance formed at the interface between the adhering microglia and/or astrocytes and the PPMP platform rather than across the scar tissue. These observations suggest that the prevention of both astrocytes and microglia adhesion to the electrodes is required to improve FP recording quality and yield.

## Introduction

Electrophysiological recordings and the stimulation of neurons by *in vivo*-implanted multielectrode arrays (MEA) is the gold standard in brain research and clinical applications (Lebedev and Nicolelis, 2017; Thompson et al., 2020). Implanted MEA platforms enable parallel extracellular field potential (FP) recordings generated by hundreds of individual neurons at high temporal and spatial resolution (Jun et al., 2017; Raducanu et al., 2017; Steinmetz et al., 2018; Gulino et al., 2019). Various types of MEA implant technologies also serve to stimulate specific brain regions to ameliorate neurodegenerative, motor, and psychiatric disorders (Gulino et al., 2019; Lozano et al., 2019) and replace damaged sensory inputs, such as in retinal implants (Bloch et al., 2019). Parallel recording of FPs from non-human primates and humans are also used to record FP patterns that can be harnessed to control robotic arms (Schwartz, 2004; Ajiboye et al., 2017; Lebedev and Nicolelis, 2017).

In spite of the immense interest, importance, and progress in bioengineering of *in vivo* brain implants, their use is severely limited by their small signal-to-noise ratio and the deterioration of their recording quality and yields (Biran et al., 2005; Salatino et al., 2017a). Aside from mechanical failures (Jorfi et al., 2015; Kozai et al., 2015a), brain implants initiate and perpetuate local inflammatory processes which culminate in the formation of encapsulating scar tissues around the implant. This foreign body response (FBR) obstructs recording and stimulation qualities (Gray et al., 1995; Rousche and Normann, 1998; Kipke et al., 2003; Nicolelis et al., 2003; Wellman et al., 2019). The prevailing views attribute these obstructions to the convergence of three main mechanisms: (I) the displacement of neurons away from the electrode surface by the progressive increase in the thickness of the glial scar (Polikov et al., 2005; Salatino et al., 2017a); (II) insulation of the electrodes from the neurons by the high electrical resistivity of the scar tissue and a self-assembled biofouling layer at the electrode surface (Johnson et al., 2005; Polikov et al., 2005; Otto et al., 2006; Williams et al., 2007; Sommakia et al., 2009, 2014; Prasad and Sanchez, 2012; Malaga et al., 2016); (III) reduced excitability, synaptic connectivity (Vezzani and Viviani, 2015; Salatino et al., 2017b, 2019), and demyelination (Winslow and Tresco, 2010; Winslow et al., 2010) of the neurons in the vicinity of the implant.

The initiation, perpetuation, and severity of the inflammatory processes induced by MEA implants depend on a large number of factors including the material from which the platform is constructed, its flexibility, the platform’s footprint, and its microarchitecture (for reviews, see Wellman et al., 2019; Thompson et al., 2020). Aside from the ultra-small and ultra-flexible family of MEA implants which appears to seamlessly integrate with the brain parenchyma (Kozai et al., 2012a; Xiang et al., 2014; Fu et al., 2016; Luan et al., 2017; Zhao et al., 2017; Zhou et al., 2017; Wei et al., 2018; Guan et al., 2019; Hong and Lieber, 2019; Yang et al., 2019), the inflammatory cascades initiated by standard cortical MEA platform implantations progresses in a comparable manner. Breaching of the blood–brain barrier (BBB) and damage to tissues along the path of the implantation leads to leakage of blood proteins, infiltration of blood-borne immune cells into the brain’s parenchyma, and a transient change in the extracellular ion concentration (Winslow and Tresco, 2010; Winslow et al., 2010; Saxena et al., 2013). These proteins bind to the implant and promote activation of the microglia (Bjornsson et al., 2006). ATP and danger-associated-molecule patterns (DAMPs) released from the injured and degenerating cells are also recognized by the microglia, the resident macrophages of the CNS, that serve as the first line of defense against foreign materials and different forms of sterile mechanical trauma (Beg, 2002; Anderson et al., 2008; Kozai et al., 2012b; Potter et al., 2013; Hermann and Capadona, 2018; Roh and Sohn, 2018). Live two-photon microscope imaging (Kozai et al., 2012b, 2014a, 2016a, b; Eles et al., 2017, 2018; Wellman and Kozai, 2018) has documented that minutes after implantation of a MEA platform into mouse cerebral cortex, microglia residing at a distance of approximately 100 μm from the implant surface sense and respond to the damage by directing processes toward the implant. Within 6–24 h after implantation, the activated microglia migrate toward the implanted platform surface. Three days post-implantation, almost 100% of the implant is surrounded by the activated microglia (Kozai et al., 2014a; Eles et al., 2017; Wellman and Kozai, 2018). The microglia activated by the MEA implant become phagocytic and secrete proinflammatory cytokines, chemokines, and reactive oxygen intermediates leading to neurodegenerative processes (Salatino et al., 2017a).

Thus overall, consistent with the vast literature on traumatic brain injuries (TBI) and spinal cord injuries (SCI), the activation of microglia by MEA platform implantation temporally precedes the activation of astrocytes (Polikov et al., 2005; Huang et al., 2020) and spatially borders the injury site or the MEA implant (Polikov et al., 2005; Kozai et al., 2012b; Potter et al., 2013; Campbell and Wu, 2018; Huang et al., 2020).

Understanding the spatiotemporal relationships between cells that induce and perpetuate the structure and function of the inflammatory FBR in response to MEA platform implantation is fundamental to the development of effective approaches to mitigate these processes. Notable progress has been made in understanding the complex cell biological mechanisms underlying the inflammatory response (Zhang et al., 2010; Kozai et al., 2015b; Hermann and Capadona, 2018). Nonetheless, the relative roles of microglia and astrocytes in the orchestration of the encapsulating scar are not well understood. For example, to what extent does reciprocal signaling between the early activated microglia and the delayed astrocytes play a role in defining the implant-encapsulation process? Would microglia dilution/depilation lead to accumulation of cellular debris around the MEA implant and hence to augmented inflammation? Could astrocytes, in the absence of microglia, respond to MEA platform implantation by forming an encapsulating scar around the implant? In the context of the recording and stimulation of neurons by implanted MEA, would changes in the bulk cellular composition of the encapsulating scar alter the functional qualities and yield of the implanted MEA platforms? Addressing these questions is important not only from a basic brain research point of view but would also contribute to the logical design of solutions to overcome the barriers that undermine the effective use of MEA implants for research and clinical applications.

The recent discovery that the survival of microglia in adult brains depends on the expression of the colony-stimulating factor 1 receptor (CSF1R) and the development of brain permeant CSF1R inhibitors (Patel and Player, 2009; Elmore et al., 2015) has enabled the scientific community to start examining the roles of microglia under normal conditions and in different types of brain pathologies and trauma. Inspired by these studies, here we begin to investigate the role of microglia in triggering and orchestrating the inflammatory cortical brain response initiated by MEA platform implantation.

Colony-stimulating factor 1 receptor is a cytokine receptor which is highly expressed by the microglia of adult rodents (Elmore et al., 2014, 2015). Inhibition of the CSF1R in adult mice using PLX3397 or PLX5622 chow leads to a loss of 90–95% of the microglia within 5–7 days of feeding mice with PLX 5622 chow (Goldmann et al., 2013; Bruttger et al., 2015; Dagher et al., 2015; Spangenberg et al., 2019). Interestingly, the peripheral macrophages and other myeloid cells are less receptive to PLX5622-mediated depletion (Mok et al., 2014; Spangenberg et al., 2016 but see Lei et al., 2020). Despite the robust depletion of the brain’s microglia, the motor skills and cognitive functions of the mice depleted of microglia by PLX5622 were not altered. Interestingly, microglia ablation in Alzheimer-diseased mice models resulted in beneficial effects in terms of dendritic spine and neuronal loss and improved contextual memory (Elmore et al., 2014; Dagher et al., 2015; Acharya et al., 2016; Spangenberg et al., 2016, 2019; Willis et al., 2020).

**FIGURE 1:**
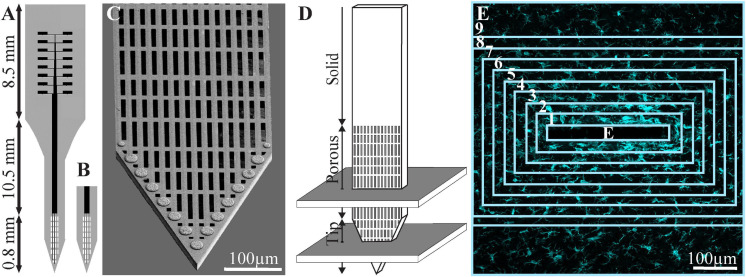
Schematic drawing of the perforated polyimide-based MEA platform (PPMP) and the orientation of the horizontal cryostat slices along with the paraformaldehyde-fixed cortical tissue around it. **(A)** A functional PPMP. **(B)** A free-floating non-functional platform. **(C)** SEM image of the perforated part of a PPMP. **(D)** Schematic illustration of the implanted part of the PPMP and the orientation of the 40-μm-thick horizontal slices along with the paraformaldehyde-fixed cortical tissue around it. A number of cortical slices were prepared from the non-functional PPMP tip and perforated parts. Ten consecutive confocal microscope optical sections were grabbed from the immunolabeled slices at intervals of 1 μm to prepare maximal projection images of the sectioned platform and the tissue surrounding it. **(E)** The immunofluorescent intensity or cell numbers within the electrode (central rectangle) and within nine 25-μm-wide centripetal shells around it, were measured or counted and processed to establish the normalized fluorescent intensity level (NFI) or cell-type densities, at a given distance around the electrode. The image in panel **(E)** depicts an example microglia labeled by Iba-1.

Using PLX5622 chow and a relatively large footprint perforated polyimide-based multielectrode array platform (PPMP) (Figure 1) which can be thin sectioned for immunohistological examinations along with the surrounding tissue (Huang et al., 2020), we took a basic-research perspective to investigate the prevailing concept that microglia, “the professional phagocytes of the CNS” and a major source of proinflammatory cytokines and chemokines, serve critical downstream functions in sensing and translating damage by MEA platform implantation to what appears to be an orchestrated inflammatory FBR cascade. Specifically, we examined how inflammatory FBR induced by MEA platform implantation develops in rat cortices depleted of 89–94% of their microglia population by the brain permeant CSF1R inhibitor PLX5622.

## Materials and Methods

### Animals and Drug Administration

All the procedures in the present study were approved by the Committee for Animal Experimentation at the Institute of Life Sciences of the Hebrew University of Jerusalem. All procedures were carried out in accordance with the approved guidelines. Both immunohistological and functional recordings were conducted using female Sprague Dawley rats (240–340 g). For microglial ablation, the rats were fed a PLX5622 diet (1200 PPM PLX5622, Plexxikon Inc., Berkeley, CA, United States) *ad libitum*. The control diet consisted of the same basic formula. PLX5622 was provided by Plexxikon Inc. and formulated in AIN-76A standard chow by Research Diets Inc. at PPM.

### Neuro-Implants

For this study, PPMPs were engineered in-house using standard photolithography fabrication methods as detailed earlier by our laboratory (Huang et al., 2020). Two types of single-shaft, 280-μm wide, and 16-μm-thick platforms carrying 15 planar gold electrodes were used, as follows: (a) non-functional platforms for the immunohistological studies and (b) functional platforms for the electrophysiological recordings. Whereas the functional platforms were terminated by an output pad at the proximal end of the shaft, the non-functional platforms were fabricated without an output pad (Figure 1). The tip of both the functional and the non-functional MEA platforms were divided into two segments: a 0.8-mm-long perforated distal part, and a 10.5-mm-long proximal solid shaft. The perforated segment tapered to form a sharp tip. The width of all the rectangular perforations (for short perforations) was 7–8 μm, and the lengths of the different perforations were 65, 47, and 44 μm (Figure 1; Huang et al., 2020).

To mechanically stabilize the functional PI platform for implantation, the output pad area and a 6.8-mm-long segment of the PI shank were glued to a 300-μm-thick Kapton tape leaving the most distal 1.3–2 mm PI shank free for insertion (Huang et al., 2020).

### Implantation of Functional Electrodes

The implantation of the functional PPMP for chronic wireless electrophysiological recordings in freely moving rats was divided into two stages: (1) preparation of a base for the platform implant, and (2) 14–28 days later, implantation of the recording platform into the brain tissue. The time lag between the invasive parts of the surgery (1) and the very delicate part of platform implantation (2) allowed us to start the wireless electrophysiological recordings soon after the PPMP implantation in the freely behaving animals in which the major tissue damage inflicted in stage (1) had completely healed (Huang et al., 2020).

### Stage 1, Base Preparation

Female rats (240–340 g) were initially anesthetized in an induction chamber with sevoflurane (8% in air, Piramal Critical Care Inc., Bethlehem, PA, United States) using a SomnoSuite^®^ Low-Flow Anesthesia System (Kent Scientific Corporation, Torrington, CT, United States). The head was shaved, and the rat was placed in a stereotaxic instrument with a mask for gas anesthesia (David Kopf Instruments, Tujunga, CA, United States). The sevoflurane concentration was slowly adjusted to a level of about 3.7% and maintained at this level throughout surgery (Polterovich et al., 2018). The surgical level of anesthesia was verified by a lack of pedal-withdrawal reflex and breathing rate. Body temperature was controlled with a closed loop heating system with a rectal probe. The eyes were protected with a thick layer of Vaseline, and the skin on the head was disinfected with povidone-iodine solution (10%, equivalent of 1% iodine, Rekah Pharm., Ind., Ltd., Holon, Israel). To prevent post-operative pain, the rats received a subcutaneous injection of carprofen 50 mg/ml (5% W/V, Norocarp, Norbrook, Newry, Northern Ireland, United Kingdom) using a dose of about 12 mg/kg during surgery.

A 1.5–2-cm longitudinal cut of the skin on the head was made, and the dorsal surface of the skull was exposed. The connective tissue covering the bones was removed, and the bones were cleaned with sterile saline. The surface of the bones was then treated with a 15% hydrogen peroxide solution (Sigma-Aldrich Inc., St. Louis, MO, United States), and the area was flushed with sterile saline after 10–20 s. When the surface of the skull was clean and dry, a reference point for the entrance of the recording electrodes was marked to target the motor cortex at the following coordinates: AP: +3.5 to +5.0 mm; ML: +2.5 mm from the Bregma (Barthas and Kwan, 2017; Ebbesen et al., 2018). Subsequently, six openings for supporting screws were drilled and screws were mounted in the skull. One screw soldered to a ground wire was placed in the left frontal bone. The screws were fixed together and to the bone first with resin and then with acrylic dental cement (Super-bond C&B, Sun Medical, Moriyama, Shiga, Japan; Coral-Fix, Tel Aviv, Israel) to form the base of the implant. The ground wire was connected to a small female connector (853 Interconnect Socket, MILL-MAX MFG. Corp., Oyster Bay, NY, United States) embedded in the cement in the front of the implant base to create an easy, low impedance connection to the ground wire during recordings. A thin polyimide tube was placed on the skull vertically above the projected electrode implantation site and cemented together with the rest of the implant base.

The wounds were cleaned and treated *in situ* with antibiotic ointment (synthomycine, chloramphenicol 5%, Rekah Pharm. Ind. Ltd., Holon, Israel) and Bismuth subgallate (Dermatol, Floris, Kiryat Bialik, Israel). The skin was sutured (Nylon, Assut sutures, Corgémont, Switzerland) in the anterior part of the implant with one or two sutures to stretch the skin around the base of the implant. Rats received an intraperitoneal injection of enrofloxacin antibiotic 50 mg/ml (5% W/V) at a dose of 15 mg/kg diluted with saline for a total volume of 1 ml (Baytril, Bayer Animal Health GmbH, Leverkusen, Germany). For the first 2 days after surgery, meloxicam (Loxicom 1.5 mg/ml, Norbrook, Newry, Northern Ireland, United Kingdom) was dissolved in palatable wet food and served to the rats in their home cages (0.6 mg in one portion given every 24 h). Rats were allowed at least 2–4 weeks of recovery post-surgery before starting the next procedure. After surgery, the animals were housed individually to prevent damage to the implants.

### Stage 2, Platform Implantations

The anesthetic procedure toward this stage commenced as described for stage 1. Thereafter, the dental cement above the implantation site marked by the polyimide tube was removed using a dental drill until the skull was exposed. The craniotomy was performed by drilling, and the dura was gently resected (0.3–0.5-mm-long incision). Electrodes were inserted into the brain tissue using a micromanipulator (to a depth of 1.1–1.5 mm below the brain surface) at a rate of 100 μm/min. Neural activity was monitored during insertion to identify the optimal depth. The craniotomy was sealed with elastic silicone polymer (DuraGel, Cambridge Neurotech, United Kingdom), and the electrodes were fixed to the base of the implant with acrylic dental cement.

To prevent post-operative pain, the rats received a subcutaneous injection of carprofen 50 mg/ml (5% W/V) at a dose of about 12 mg/kg. For the first 2 days after surgery, meloxicam dissolved in palatable wet food was served to the rats in their home cages (0.6 mg in one portion given every 24 h).

### Implantation of PPMP Platforms for Immunohistological Studies

For economic and technical reasons related to the cryosectioning of the skull and the screws, we used non-functional platforms for the immunohistological studies. These platforms were implanted into the motor cortex (coordinates: AP: +3.5 mm; ML: +2.5 mm from the Bregma) without fixing (gluing) the electrode to the skull, as detailed above (Huang et al., 2020).

### Non-functional Electrode Platform Implantations

A 1–1.5-cm longitudinal cut of the skin on the head was made, and the anterior and dorsal surfaces of the skull were exposed. Two craniotomies, one in the left and the other in the right frontal bones, were performed at the desired reference points and the dura was gently resected (0.3–0.5-mm-long incision). The 1.7-mm-long platforms (only the shank without the pad output, Figure 1) were slowly inserted into the motor cortex. The electrodes were released from the holder, and the craniotomy was sealed with melted bone wax (W810, Ethicon, Belgium). The wound was treated *in situ* with antibiotic ointment (synthomycine, chloramphenicol 5%) and sutured with nylon sutures. The rats received an intraperitoneal injection of enrofloxacin at 50 mg/ml (5% W/V) and meloxicam in palatable wet food.

### Electrophysiology

Voltage recordings from freely moving rats were amplified and digitized using a 32-channel multichannel system wireless amplifier (W2100-HS32 Multichannel systems, a division of Harvard Bioscience, Inc.) connected to the PPMP by 16 channel Omnetics connectors (A79042-001). As a ground reference, we used the screws attaching the MEA platform to the skull (Precision Technology Supplies Ltd., United Kingdom). The sampling rate was 20 kHz, and a 5-Hz high-pass and a 3,000-Hz low-pass filter were applied for local field potential (LFP) and single-unit recordings. Spike sorting was performed using the fully automatic spike-sorting implementation described in Chaure et al. (2018). Electrode impedances were measured *in vitro* before implantation and *in vivo* at 1 kHz using the nanoZ impedance tester (Plexon).

### Tissue Processing for Immunohistology

Rats implanted with non-functional MEA platforms were killed at 1 h, 1 and 3 days, and 1, 2, 4, and 8 weeks after implantation. For brain tissue fixation, individual rats were deeply anesthetized with isoflurane (Piramal, United States) followed by an IP overdose injection of pentobarbital (4.5 ml/250 g rat, CTS Group, Israel). When breathing had stopped, the rats were transcardially perfused with phosphate buffer saline (PBS). This was followed by a 4% paraformaldehyde in PBS (PFA, Sigma-Aldrich) perfusion at a rate of 10 ml/min for 40 min.

Next, the skulls were removed and the implanted brain was post-fixed at 4°C for an additional 12–24 h in PFA. Thereafter, the fixed and exposed brains were washed in PBS and incubated for 1–3 days in a 30% sucrose solution in PBS at 4°C.

### Cryosectioning and Immunohistology Labeling

To prepare for cryosectioning of the brain tissue, a cubic-shaped portion of tissue, (approximately 1 × 1 × 1 cm) with the perforated PI platform in its center was isolated. The isolated piece of brain was placed in a freezing medium (Tissue-Plus O.C.T. Compound, Scigen) and frozen at −80°C. The frozen tissue along with the implanted platform was then horizontally sectioned into 40-μm-thick slices using a Leica CM1850 Cryostat. Individual slices were collected and placed in 24-well plates containing PBS.

The tissue slices were then incubated in blocking solution [1× PBS, 1% heat-inactivated horse serum (Biological Industries), 0.1% Triton X-100 (Sigma-Aldrich)] for 1 h at room temperature (RT) under gentle shaking. Next, the slices were incubated with a diluted primary antibody for 3 h at RT and washed three times with the blocking solution. This was followed by 1-h incubation at RT with the diluted secondary antibody after which the slices were washed with the blocking solution three times and stained with the nuclear marker DAPI (Sigma-Aldrich, 1 mg/ml 1:1,000) for 15 min at RT. After washing with the blocking solution and PBS, the slices were mounted on Superfrost Plus Slides (Thermo Fisher Scientific) and sealed by a Vectashield (VE-H-1000-Vector Labs) mounting medium. Meticulous examination of the prepared tissue slices by confocal microscope optical sections revealed that the antibodies penetrated the tissue to homogeneously stain the target cells.

Neurons were concomitantly labeled with two antibodies: one for neurite labeling [mouse anti-Neurofilament 160/200 monoclonal antibody (Sigma-Aldrich N2912, 1:10,000–1:20,000)] and the other for neuronal nuclei [mouse anti-NeuN monoclonal antibody (Merck MAB377, 1:200)]. Astrocytes were labeled with chicken anti-glial fibrillary acidic protein (GFAP) polyclonal antibodies (Thermo Fisher PA1-10004, 1:500–1,000). Microglia were labeled using rabbit anti-Ibl-1 monoclonal antibody (Abcam ab178846, 1:1,000). For the secondary antibodies, we used goat anti-mouse Alexa 488, goat anti-chicken Alexa 647 (Thermo Fisher A-11001 and A21449, respectively, 1:100), and sheep anti-rabbit Cy3 (Sigma-Aldrich C2306, 1:100). To confirm that the Iba-1 antibody labeled microglia and did not infiltrate the macrophages, we co-labeled the Iba-1-positive cells using goat anti-Iba-1 antibody (Abcam ab5076, 1:100–125) with rabbit polyclonal antibody for rat TMEM119 that recognizes microglia-specific transmembrane proteins (Synaptic Systems GmbH, 1:100, 400 203). To co-label the microglia and the dividing cells, we used goat anti-Iba-1 (Abcam ab5076, 1:100–125) together with rabbit anti-polyclonal KI67 (Abcam, ab15580, 1:200). For the secondary antibodies, we used donkey anti-goat IgG H&L Alexa405 (Abcam ab175665, 1:100) and sheep anti-rabbit Cy3 (Sigma-Aldrich C2306, 1:100).

### Microscopy

Confocal image stacks of the immunolabeled slices were acquired with an Olympus FLUOVIEW FV3000 confocal scan head coupled to an IX83 inverted microscope, using a ×20 air objective (NA = 0.75). Scanning was done in sequential mode in two phases. In the first phase, DAPI was excited using the 405-nm laser, and its emission was acquired in the 415- to 470-nm range using a spectral detector, whereas Cy3 was excited using the 561-nm laser and its emission was acquired in the 570–630-nm range using a second spectral detector. In the second phase, Alexa488 was excited with the 488-nm laser and its emission collected in the 500- to 540-nm range, whereas the Alexa647 was excited using the 640-nm laser, and its emission was collected in the 645- to 745-nm range, again using the two spectral detectors. A non-confocal transmitted light image was also acquired and served to visualize the electrode.

For the co-staining of Iba-1 and TMEM-119 or Iba-1 and KI67, we used the sequential mode. Alexa405 was excited using the 405-nm laser, and its emission was acquired in the 420- to 495-nm range using a spectral detector, whereas Cy3 was excited using the 561-nm laser and its emission was acquired in the 575–650 nm range using a second spectral detector. A non-confocal transmitted light image was also acquired and served to visualize the electrode. Typically, 15–30 confocal slices were acquired, with a vertical spacing of 1 μm. Image stacks were acquired from two regions of the electrode: tip (T), and porous (P).

### Image Processing, Analysis, and Statistics

The image processing was implemented using the Fiji distribution of ImageJ (Schindelin et al., 2012; Schneider et al., 2012), as follows. A maximum intensity projection image was created using 10 consecutive optical sections from each of the 40-μm-thick brain slices that were prepared. A rolling ball filter with a radius of 50 pixels was applied to the maximum intensity projection images to remove the background. This was implemented using the subtract background function in ImageJ. A region of interest (ROI) that defines the electrode was manually created. A set of concentric ROIs was automatically created around the electrode, where each ROI had a width of 25 μm.

Two methods of analysis and representation of the cell densities in contact and around the PPMPs were used:

(1) The densities of the astrocytes and neurons, including their cell bodies and neurites, were analyzed and depicted as the relative fluorescent intensities with respect to the normal background; these are referred to as the NFI values. To that end, the average fluorescence intensity signal (of either the astrocytes or the neurons) within each ROI was measured. That same concentric ROI set was used on the corresponding control section in which there was no electrode, taken >300 μm away from the implant. The normalized fluorescence distribution maps were created by dividing the value of the mean gray level of a given ROI (shell) from the image with the electrode by the value from the corresponding ROI in the control.

(2) The NFI approach could not be applied to compare the microglia densities between the control rats and the PLX5622-treated rats since the PLX5622 treatment reduced the cortical microglia densities to 6–11% vs. the controls. Therefore, the denominators to calculate the NFI (fluorescent intensity near the implant/fluorescent intensity remote from the implant) of the control and PLX5622-treated rats could not be compared. For that reason, the density values of microglia in the present study (for both the control experiments and the PLX5622 treated rats) were calculated by counting the numbers of microglia. The microglia densities are given here as the average number of cells per 100 μm^2^ at a given shell. To count the microglia, we used merged images of Iba-1-labeled cells complemented by DAPI labeling. The microglia nuclei were unequivocally identified using the characteristic heterochromatin distribution, nuclear shape, and size (Huang et al., 2020).

Association of a cell that crossed the borderline between two ROI (shells) to a given shell was decided in accordance to the largest fraction of its immunolabeling. If the center of the immunolabeled portion coincided with the border line between two shells, the cell was counted as residing in the inner shell. It is noted that microglia that adhered to the PPMP surface (not the pores) were counted as residing in the first shell (0–25 μm).

A similar approach was used to define the density distribution of the neuronal cell bodies (in contrast to the cell bodies and neurites together). The neuronal somata were identified by co-labeling cells by NeuN that specifically stains neuronal cell bodies and DAPI. Neuronal nuclei labeled by DAPI are identified by their typical heterochromatin distribution, nuclear shape, and size as described in a previous publication from our lab (Huang et al., 2020).

Average fluorescent values and cell counting characterizing the FBR in space and time were measured and calculated from cortical brain slices prepared from 2 to 10 hemispheres/experiments at different time points (1 h, 1 and 3 days, and 1, 2, 4, and 8 weeks). Each brain hemisphere was used to prepare 1–5, 40-μm-thick tissue slices from the tip (T) and the porous (P) segments of the implant (Figure 1). Overall, each slice was used to prepare a single maximal projected image generated by 10 consecutive optical sections. The sample sizes of the immunohistological study includes altogether 37 controls, 32 PLX5622 pre-treated, and 20 post-treated rats as detailed in Supplementary Table 1. Differences between the cells’ density values at different distances from the implant and at different points in time were assessed by a *t* test for two samples assuming unequal variances. For all tests, a *p* value < 0.01 indicated a statistically significant difference (Supplementary Table 2).

Since the tissue for cross cryosections could not always be aligned perfectly, the apparent width of the PI platforms in the projected images could appear to be larger than 16 μm. As a result of the platform tilt, asymmetric cell and nucleus distributions around the electrode were sometimes observed.

## Results

### Time Course of Rat Cortical Microglia Ablation by the CSF1R Inhibitor PLX5622

**FIGURE 2:**
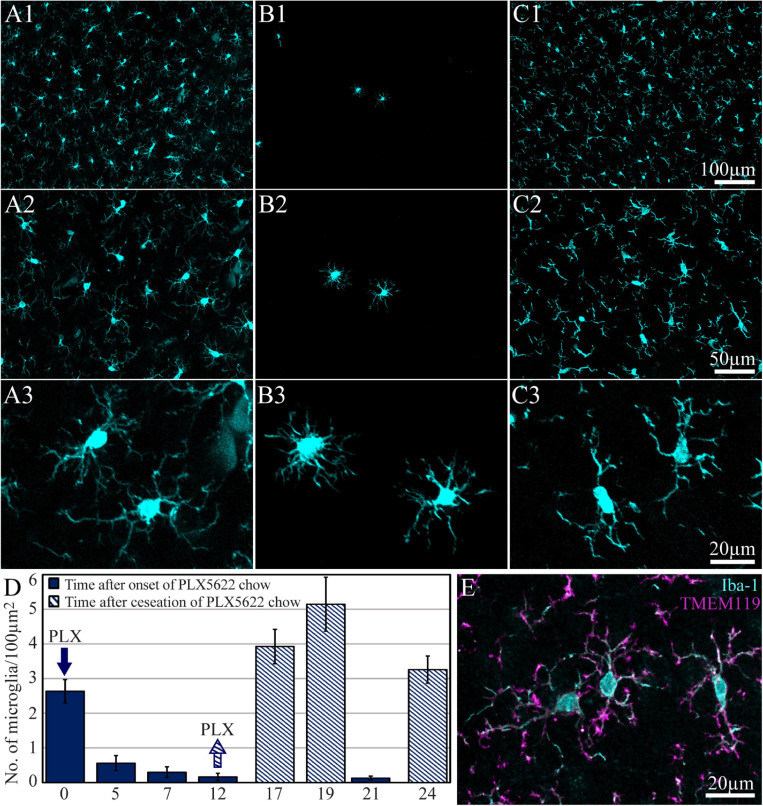
The time course of cortical microglia elimination and repopulation in rats fed *ad libitum* by PLX5622-chow. **(A–C)** Horizontal cortical sections, at three magnifications of Iba-1-labeled microglia. **(A1–A3)** The control cortex (not fed by PLX5622), **(B1–B3)** the cortex of a rat fed with PLX5622 for 12 days, and **(C1–C3)** the cortex of a rat that was fed for 12 days with PLX5622 and then switched back to a normal diet for 7 days. **(D)** The time course of microglia elimination by PLX5622 feeding (blue columns) and recovery after switching back to the control diet (diagonal stripes). **(E)** Colabeling of microglia by Iba-1 (cyan) and TMEM 119 (magenta).

A large body of studies has established that the CSF1R inhibitor PLX5622 eliminates approximately 95% of the microglia from the adult mouse CNS. Upon withdrawal of the drug, the microglia population recovers with no contribution from non-microglial lineages (Elmore et al., 2014, 2015; Bruttger et al., 2015; Spangenberg et al., 2019; Zhan et al., 2019). Importantly, it was shown that whereas PLX5622 chow efficiently depletes microglia in mouse brains, it has no effect on the peripheral populations of monocytes/macrophages (Elmore et al., 2014, 2015; Bruttger et al., 2015 but see Lei et al., 2020). To the best of our knowledge, no parallel reports are available on the effects of PLX5622 chow on rat microglia. Recently, however, Riquier and Sollars (2020) demonstrated that intraperitoneal injections of PLX5622 deplete rats’ microglia in the brainstem. Therefore, we began this study by determining the time course of microglia elimination and repopulation from female Sprague Dawley rats’ cerebral cortices fed *ad libitum* with PLX5622 chow. The observations revealed that female rats fed with PLX5622 chow behaved and gained weight as controls for periods of 8 weeks (as long as most experiments were conducted and for longer times of 4 months in a number of cases). To characterize the time course of microglia elimination and repopulation with PLX5622 chow, we first established the microglia density in intact cortices (not implanted by MEA platforms) by immunolabeling microglia with Iba-1 (Figure 2). Similar to earlier reports on the mouse CNS (for example, Zhan et al., 2019) within 5 days of feeding with PLX5622 chow, the density of the microglia dropped to 21.3 ± 8.2% from normal and then further declined to 6.2 ± 3.9% and 4.9 ± 2.1% between days 12 and 21. Importantly, as in mice, approximately 5% of the cortical microglia population resisted the CSF1R inhibition by PLX5622 (Rice et al., 2015; Spangenberg et al., 2016). Switching back from PLX5622 diet to control chow led to repopulation of the cortical microglia (Figure 2).

Based on the time course of microglia elimination from rat cortices by PLX5622, we next examined whether depletion of microglia by PLX5622 chow affected the characteristic immunohistological inflammatory response to PPMP implantation. To that end, we compared the spatiotemporal distribution patterns of microglia, astrocytes, and neurons following PPMP implantation using three experimental protocols: (a) control: a control diet fed to rats implanted by PPMPs. (b) Rats in which the PPMP implantations to cortices was preceded by 7–10 days of PLX5622 feeding. PLX5622 chow feeding was continued uninterruptedly for up to 8 weeks after the PPMP implantation. (c) The onset of PLX5622 chow feeding began 3–4 days after PPMP implantation to intact cortices and continued for up to 8 weeks thereafter.

### Microglia Inflammatory Cascades After PPMP Implantation in Control and PLX5622-Treated Rats Control

**FIGURE 3:**
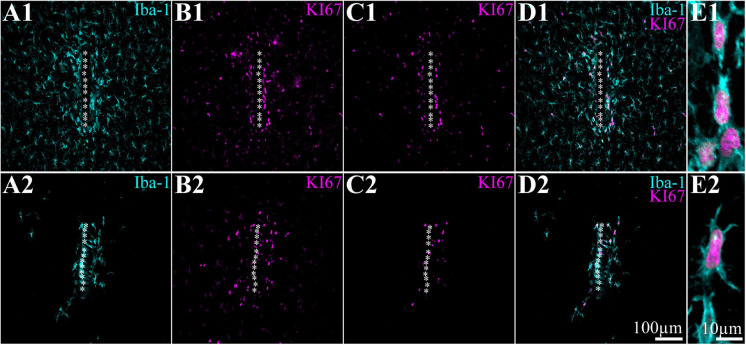
PPMP implantation induces mitosis of microglia in close proximity to the platform and in contact with the implant but not farther away from it. Shown are horizontal sections through the perforated segments of PPMPs implanted for 5 days. For purposes of orientation, the solid PI “ridges” in between the pores of the PPMP are labeled with white asterisks. **(A1–E1)** Control, **(A2–E2)** sections from rats fed with PLX5622 chow for 7 days before PPMP implantation and onward. **(A1,A2)** Iba-1-labeled microglia. **(B1,B2)** KI67-labeled microglia and unidentified cells. **(C1,C2)** KI67-labeled microglia only defined by co-labeling of Iba-1 and KI67 (see **D1–E2**). **(D1,D2)** Merged images shown in panels **(A1,C1)** and **(A2,C2)** correspondingly. **(E1,E2)** Enlarged merged Iba-1 (cyan) and KI67 images (magenta).

**FIGURE 4:**
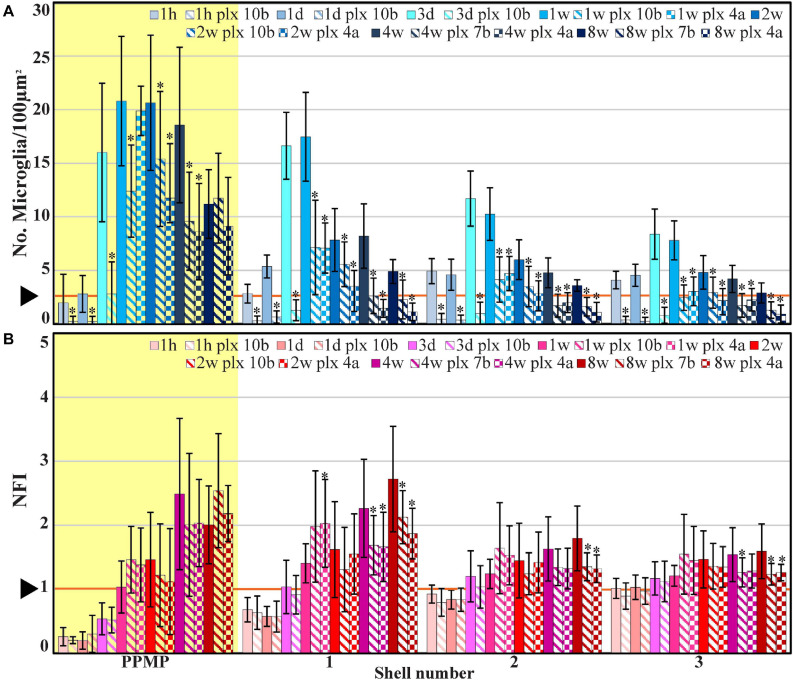
The average densities of microglia (**A**, number of cells/100 μm^2^, blue) and the normalized fluorescent intensity (NFI) of astrocytes (**B**, red) within the perforated segment of the implanted PPMPs (yellow background), in contact with the PPMP (shell 1) and in two shells around it at different time points post-PPMP implantation. Homogeneous columns depict control cortices; diagonal stripes depict cortices from rats fed with PLX5622 7–10 days before PPMP implantation and thereafter; checkered columns depict cortices of rats fed with PLX5622 3–4 days after PPMP implantations and thereafter. The times post-platform implantation (1 h, 1 and 3 days, and 1, 2, 4, and 8 weeks) are coded by the darkening of the column color as indicated by the legend on top of the histograms. The distance of the measured averages from the MEA platforms is given by the shell number. Each shell is 25 μm wide (as illustrated in Figure 1). Vertical lines correspond to one standard deviation. The orange line pointed by an arrowhead depicts the average number of microglia/100 μm^2^ in control cortices **(A)** and the normal NFI value of control astrocytes **(B)**. Asterisks indicate significant differences of *P* < 0.01 between the value of the PLX5622-treated rats and the controls at the same point in time. Similar data for the tip segment of the PPMP are shown in Supplementary Figure 1. For the complete astrocyte NFI density map and average microglia densities of the nine shells around the implant, see Supplementary Figure 2.

**FIGURE 5:**
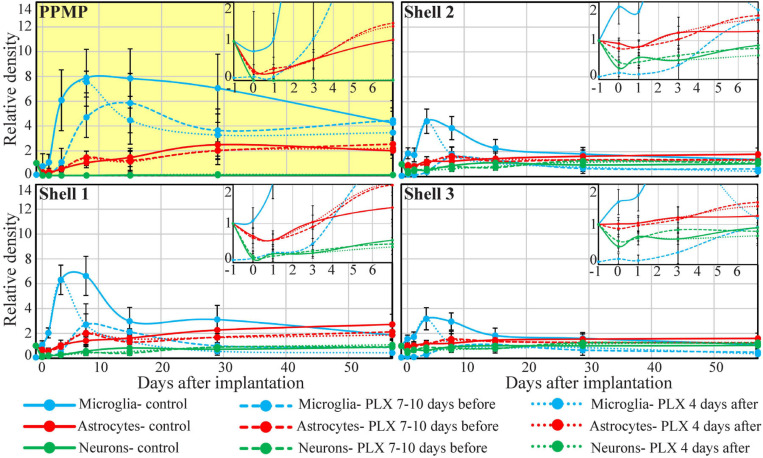
Time course of the relative changes in microglia, astrocyte, and neuron densities (yellow background), in shell 1 (which includes cells adhering to the PPMP surfaces) and shells 2 and 3. Microglia (blue), astrocytes (red), and neurons (green). Control observation (solid lines); dashed lines depict cortices from rats fed with PLX5622 7–10 days before PPMP implantation and thereafter, and the dotted lines depict cortices of rats fed with PLX5622 3–4 days after PPMP implantation and thereafter. The curves inserted on the top right hand side are expansions of the values between the control (before implantation, –1) and 7 days post-PPMP implantation. The lines were fitted using the “scatter with smooth line” application (Excel).

In a recent study, our laboratory (Huang et al., 2020) characterized the inflammatory scar formation around implanted PPMPs. Briefly, we reported that PPMP implantation to control cortices initiates the following immunohistological spatiotemporal cascade: (I) within an hour to a day post-implantation, the platform is surrounded by an acellular “shell” (edema, Huang et al., 2020). (II) Consistent with earlier live two-photon microscope imaging (Kozai et al., 2012b, 2014a, 2016a, b; Eles et al., 2017, 2018; Wellman and Kozai, 2018), microglia at a distance of 50–100 μm away from the implant surface “sense the implantation” as indicated by the orientation and extension of their branches toward the implant. (III) Three days post-PPMP implantation, shells 1 and 2 (0–50 μm) around the implant fill up with activated amoeboid-shaped microglia that adhere to the PPMPs’ surfaces and invade the platform’s pores (Huang et al., 2020). (IV) Co-labeling of cells by Iba-1 and TMEM 119 (an antibody that recognizes specific rat microglia transmembrane proteins) confirmed that the Iba-1-labeled cells were genuine microglia rather than other types of myeloid-derived cells (Figure 2E; Huang et al., 2020). (V) Co-labeling of microglia by Iba-1 and KI67 (which labels dividing nuclei) showed that approximately 70% of the total dividing cells around the implant at that point in time were microglia (Figures 3A1–E1). (VI) Approximately 3–7 days post-PPMP implantation, the density of microglia within the PPMP adhering to it (first shell) and in the shells 2–9 reached a peak (Figures 4, 5 and Supplementary Figures 1, 2). (V) Thereafter, 2 week post-implantation, the density of microglia in shells 1–9 gradually recovered and the morphology of the microglia returned to the resting configuration. Importantly, the density of microglia occupying the PPMP pores and adhering to it remained higher for approximately 8 weeks as compared with the control (Figures 4, 5 and Supplementary Figures 1, 2).

### Spatiotemporal Distribution of Microglia in PLX5622 Chow Pre-fed Rats

**FIGURE 6:**
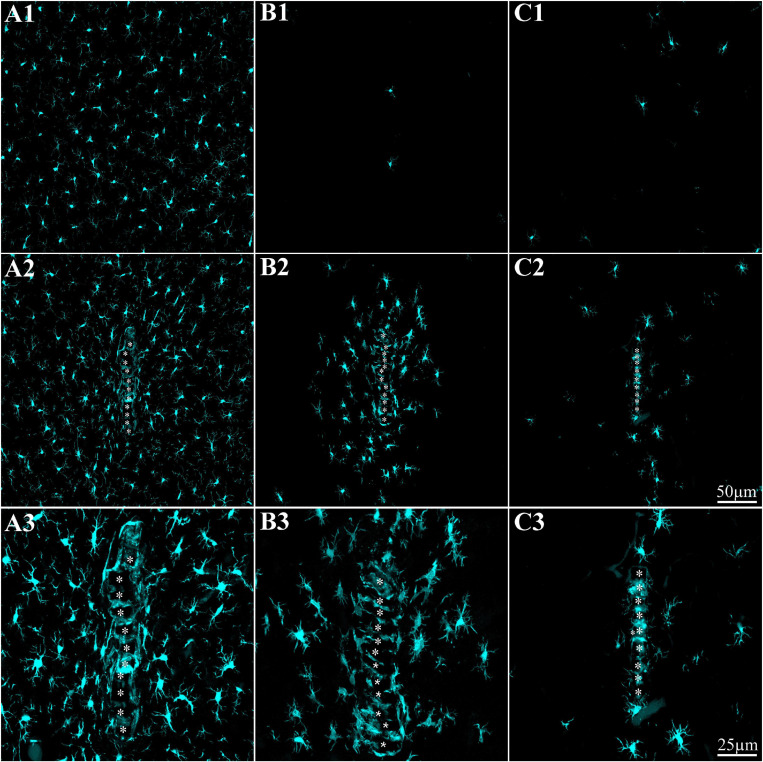
Implantation of a PPMP to cortices depleted of microglia by a PLX5622 diet induces local repopulation by PLX5622-insensitive microglia within and in the vicinity of the implant but not away from it. **(A)** PPMP implant in the control cortex; **(B)** PPMP implant in the cortex of a rat fed with PLX5622 chow 10 days before implantation and thereafter; **(C)** PPMP implant in the cortex of a rat fed with PLX5622 chow 4 days after implantations and thereafter. All shown cortices were fixed 2 weeks after the PPMP implantation. **(A1,B1,C1)** are images taken ≥200 μm away from the implants. Note the normal microglia density in the control **(A1)** and the microglia-depleted cortex of the PLX-fed rats **(B1,C1)**. For purposes of orientation, the solid PI “ridges” in between the pores of the PPMP are labeled by white asterisks **(A2,A3,B2,B3,C2,C3)**. In the control images **(A2,A3)**, microglia are distributed around the PPMP, adhere to it, and invade the pores within the platform. In contrast, in the PLX5622 pre-fed rats and rats fed continuously with PLX5622, a “cloud” of PLX5622-insensitive microglia surrounds the implant and invades the PPMP platform pores. Likewise, a microglia “cloud” of PLX5622-insensitive microglia is formed in the cortices of rats fed 4 days after implantation with PLX5622.

**FIGURE 7:**
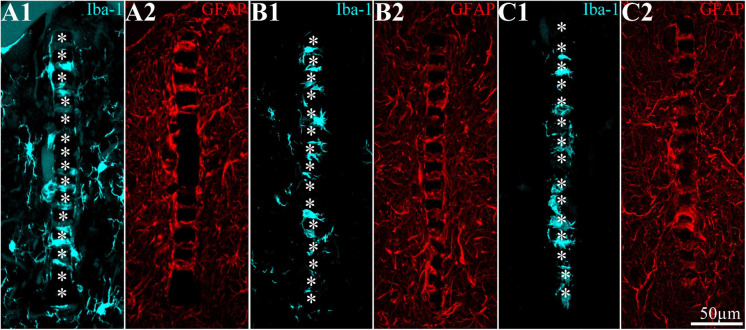
Distribution of microglia and astrocytes around and within the implanted PPMP for 8 weeks. **(A1,A2)** Control; **(B1,B2)** in a cortex of a rat fed for 7 days before implantation with PLX5622 diet and thereafter; **(C1,C2)** in a cortex of a rat fed with PLX5622 diet 3 days after implantation and thereafter. For purposes of orientation, the solid PI “ridges” in between the pores of the PPMP are labeled by white asterisks **(A1,B1,C1)**. Note that whereas in panel **(A)** the microglia (cyan) occupy the PPMP pores and the first shell around it (including in contact with the platform), in the PLX5622-treated rats **(B,C)**, the first shell is almost totally devoid of microglia. In panels **(A2,B2,C2)**, astrocytes (red) adhere to the surface of the PPMPs and occupy the first shell.

The spatiotemporal distribution dynamics of microglia following PPMP implantation to cortices of rats fed with PLX5622 chow for 7–10 days prior to PPMP implantation and continuously thereafter differed significantly from the control rats (Figures 3–6) in four main ways: (I) Whereas in the control experiments the microglia density gradually increased, reaching peak values within the PPMP pores in contact with it (shell 1) and in shells further away on days 3–7 post-PPMP implantation (Figures 4, 5 and Supplementary Figures 1, 2), in the PLX5622 pre-fed rats, the PPMP pores’ surface (shell 1) and the surrounding shells 2–9, (0–200 μm) remained almost totally free of microglia for the first 3 days post-PPMP implantation (Figures 4, 5 and Supplementary Figures 1, 2). (II) Unexpectedly as of days 5–7 after implantation, a PLX5622-insensitive, Iba-1-positive microglia population appeared to occupy the platform’s perforations, adhere to its surface (shell 1) and populated shells 1–4 (0–100 μm) (Figures 3A2–E2, 4, 5, 6B,C and Supplementary Figures 1, 2). The PLX5622-insensitive microglia density at these locations (shells 1–4) was approximately 31–41% of the control rats in the corresponding location and time (Figures 4, 5 and Supplementary Figures 1, 2). The density of PLX5622-insensitive microglia in the parenchyma around the implant (at a radius of >200 μm) was maintained at a low steady-state level of ∼5% of the control (Figures 3A2–E2, 4, 5, 6B,C and Supplementary Figures 1, 2). (III) The low-density PLX5622-insensitive microglia around the PPMP resembled a “cloud” surrounding the implant surface (Figures 3, 6). We believe that this PLX5622-insensitive “microglia cloud” was also generated in the control experiments but could not be detected against the background of the resident microglia population. (IV) The microglia densities of the control and PLX5622-treated rats declined within approximately 2 weeks of PPMP implantation. In the PLX5622-treated rats, the peak density (on week 1 post-implantation) dropped on the eighth week post-PPMP implantation from 7.1 ± 4.4 and 7.1 ± 2.3 to 2.3 ± 1.8 and 1.1 ± 0.8 cell/100 μm^2^ (in PLX pre- and post-fed, respectively). In the control groups, the decline was from 17.5 ± 4.1 to 4.9 ± 1.1 cell/100 μm^2^ (Figures 4, 5 and Supplementary Figures 1, 2). (V) Whereas the density of the microglia that occupied the PPMP perforations remained high for at least 8 weeks in the control and PLX5622 experiments (the total duration of the observations, Figures 4, 5, 7), the density of the PLX5622-insensitive microglia in the first shell (which included the microglia attached to the PPMP platform) significantly decreased in respect to the control. The absolute density of microglia in the first shell (on the eighth week post-implantation) was 2.1 and 4.3 times larger in the control group than in the pre- and post-fed PLX experiments (Figures 4, 5, 7; Supplementary Figure 2 and Supplementary Table 2).

By co-labeling the Iba-1-positive PLX5622-insensitive microglia with rat TMEM119, that recognizes microglia-specific transmembrane proteins, we confirmed that these cells were genuine microglia and not other Iba-1-positive cell types. The unexpected appearance of a PLX5622-insensitive, Iba-1/TMEM119 “microglia cloud” within the platform pores and around the PPMP implants could be due to the delayed migration of PLX5622-insensitive microglia from the parenchyma toward the implant or to the infiltration of blood-borne immune cells through the bridged BBB (Winslow and Tresco, 2010; Winslow et al., 2010; Saxena et al., 2013; Ravikumar et al., 2014; Bedell et al., 2018). Co-labeling of the PLX5622-inasensitive microglia cloud by Iba-1 and KI67 (that label cells undergoing mitosis) established that the density of the KI67-labeled microglia was significantly elevated on day 5 post-implantation within the PPMPs and around them in the PLX5622 chow pre-fed rats (Figure 3). The number of positively co-labeled microglia by Iba-1 and KI67 out of the total number of dividing nuclei accounts for the formation of the PLX5622-insensitive microglia cloud. Nonetheless, we cannot define the initial sources of the microglia that eventually form the microglia cloud around the implant. To conclude, (a) the appearance of a PLX5622-insensitive microglia population corresponded temporally to microglia mitosis, (b) these microglia began to spontaneously disappear within approximately a week after reaching a peak density, and (c) the half life time (*T*_1__/__2_) of the resting resident microglia is 7.5–15 months (Fuger et al., 2017; Tay et al., 2017; Zhan et al., 2019). These observations demonstrate that PPMP implantation triggers the appearance of a new microglia phenotype that differs from the resident microglia.

### Microglia Spatiotemporal Distribution in Rats Fed With PLX Chow 3–4 Days After Implantation

Feeding rats with PLX5622 diet 3–4 days after PPMP implantation eliminated a substantial fraction of the microglia population within 3 days of PLX chow onset (on day 7 after PPMP implantation, Figures 4–6 and Supplementary Figures 1, 2). As in the case of rats fed with PLX5622 chow before PPMP implantation, a PLX5622-insensitive microglia cloud was present around the implant and adhered to the platform surface and within the PPMP pores (Figures 4–6 and Supplementary Figures 1, 2). Since on day 7 post-PPMP implantation, and 3 days after the onset of feeding with PLX5622 chow, profiles of dividing microglia were rare (co-labeling by Iba-1 and KI67), it is reasonable to assume that the observed PLX5622-insensitive microglia cloud appeared on day 3 after PPMP implantation; that is, 1 day prior to feeding with PLX5622 chow. This interpretation is consistent with the assumption that implantation of the PPMP initiates microglia mitosis and that the daughter cells belong to the PLX5622-insensitive phenotype.

In summary, within 7–12 days, PLX5622 chow feeding eliminated 89–94% of the cortical microglia. Unexpectedly, PPMP implantation to the cortices of PLX5622-treated rats triggered a transient surge of microglia mitosis that was detected from day 5 post-implantation and onward for approximately 2 days, leading to the formation of a low-density PLX5622-insensitive microglia cloud around and within the implant. At all points in time, the density of the PLX5622-insensitive microglia surrounding the implant and adhering to it was significantly lower than that of the control rats. The density of the microglia in contact with the PPMP and in the shells around it lessened in both the control and PLX5622-fed rats; however, in rats treated with PLX5622, the density of microglia adhering to the PPMP surface (shell 1) was significantly lower than in the controls (Figures 4, 5 and Supplementaryef Figures 1, 2).

The 2-day delay in the manifestation of a PLX5622-insensitive microglia population on the implanted platform in PLX5622-treated rats and the significantly reduced microglia density in the shells around it provided the experimental background to examine to what extent activated microglia played a role in astrocyte activation and to what extent the altered microglia densities and dynamics influenced neuronal distribution and densities around the implanted MEA platform.

### Astrocytes

Numerous reports have documented that astrocyte activation by MEA platform implantation or other forms of TBI is preceded by the activation of microglia (Polikov et al., 2005; Kozai et al., 2012b; Potter et al., 2013; Campbell and Wu, 2018; Huang et al., 2020). The temporal and implied causal relationships between microglia and astrocyte activation are also evidenced by studies using PLX5622 as a way to dilute the microglia prior to damage to the CNS. For example, the transection of mouse optic nerve pre-treated by PLX5622 chow prevented the normal accumulation of microglia at the cut nerve end and delayed astrocyte accumulation by approximately 10 days (Hilla et al., 2017). Microglia depletion by PLX5622 eliminated astrocyte accumulation in response to the transection of the gustatory chorda tympani nerve of adult rats (but not in neonatal mice). PLX5622 depletion of microglia after mice SCI disrupted the formation of the glial scar (Bellver-Landete et al., 2019).

**FIGURE 8:**
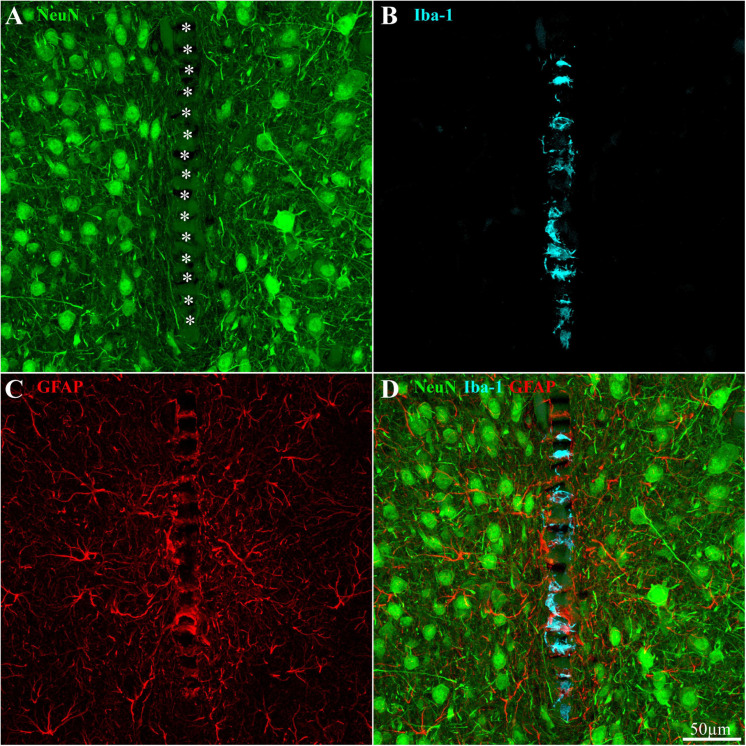
Distribution of neurons, microglia, and astrocytes around and within an implanted PPMP in a rat fed with PLX5622 diet 4 days after implantation and thereafter for 8 weeks. **(A)** Neurons (green), **(B)** microglia (cyan), **(C)** astrocytes (red), and **(D)** a merged image of panels **(A–C)**. For orientation, the solid PI “ridges” in between the pores of the PPMP are labeled with white asterisks **(A)**. Note the density of neuronal cell bodies within a distance of less than 150 μm from the PPMP platform **(A)**, the microglia mainly occupy the pores within the polyimide platform **(B)**, and the astrocyte branches adhere to the PPMP surface and invade its pores **(C)**.

Based on the observations described above, we next examined whether the elimination of 82–93% of the cortical microglia population by PLX5622 and the transient appearance of the PLX5622-resistant microglia cloud was associated with altered spatiotemporal dynamics of astrocyte activation following PPMP implantation. In an earlier study by our laboratory, we documented that the increase in astrocyte NFI levels subsequent to PPMP implantations was delayed by approximately 3 days with respect to the activation of microglia (Huang et al., 2020, Figures 4, 5 and Supplementary Figures 1, 2). Although the microglia density within the implant and in the shells around it declined from a peak value on day 7 post-implantation, the astrocyte NFI continued to increase uninterruptedly and reached a steady-state NFI level in the range of 2.7 in the eighth-week post-implantation (Figures 4, 5 and Supplementary Figures 1, 2). Co-labeling of the astrocytes by GFAP and KI67 showed that 9–15% of the total dividing cells on days 3–5 post-PPMP implantation were astrocytes. Unlike the microglia, astrocyte cell bodies were rarely seen to reside in the PPMP pores. However, astrocyte branches attached to the PPMP surface and invaded the platform’s perforations (Figures 7, 8C).

The observed astrocyte NFI level in rats fed with PLX6522 chow either 7–10 days before PPMP implantation or 3–4 days after it and onward for 8 weeks showed that despite the significant reduction in the microglia density within the PPMP and in the shells around it, the overall spatiotemporal distribution trend of astrocyte activation was not altered (Figures 4, 5; Supplementary Figures 1, 2 and Supplementary Table 2). In particular, it should be noted that as in the control rats, astrocyte activation (increased GFAP fluorescent) was delayed with respect to microglia activation by approximately 3 days post-PPMP implantation in rats fed with PLX5622 chow (Figure 4 and Supplementary Figures 1, 2). Importantly, the increase in astrocyte NFI “indifferently” progressed despite the reduced PLX5622-insensitive microglia concentration during this window of time. Nevertheless, statistical examination of the NFI values in shell 1 suggested that in PLX5622-treated rats, the astrocytic NFI was reduced on week’s 4 and 8 post-implantation to a larger extent than that of the control rats (significant level of *P* ≤ 0.005, Figure 4 and Supplementary Table 2). Despite the slight facilitation in the astrocyte-NFI recovery in the PLX5622-treated rats, the observations suggest that the typical increase in astrocyte NFI around the implants and the astrocytes’ attachment to the implant surfaces were initiated and propagated in a microenvironment of significantly reduced microglia densities. This suggests that either the molecular signaling released by the activated microglia was redundant or that proinflammatory cytokines released by the highly diluted PLX5622-insensitive microglia cloud (which developed 5 days after PPMP implantation) was sufficient to activate the astrocytes’ inflammatory cascade.

### Neurons

Multielectrode arrays-platform implantation leads to the necrosis of neurons by direct mechanical damage along the insertion path and apoptosis induced by toxic molecules released by the activated microglia and astrocytes. With time, the surviving neurons are thought to be displaced away from the platform’s surface by the progressive increases in the size of the scar tissue (Polikov et al., 2005; Salatino et al., 2017a). Furthermore, inflammatory cytokines released by microglia and astrocytes are thought to reduce neuronal excitability, disable synaptic functions, and lead to axonal demyelination (Winslow and Tresco, 2010; Winslow et al., 2010; Vezzani and Viviani, 2015; Salatino et al., 2017b, 2019). In view of the observations described above showing that the density of the PLX5622-insensitive microglia phenotype around the PPMP implant and within it was significantly smaller than in the control implants, and that the astrocyte density was slightly smaller in rats fed with PLX5622 chow, we next examined whether these alterations were associated with changes in the spatiotemporal distribution patterns of the neurons around the implants.

**FIGURE 9:**
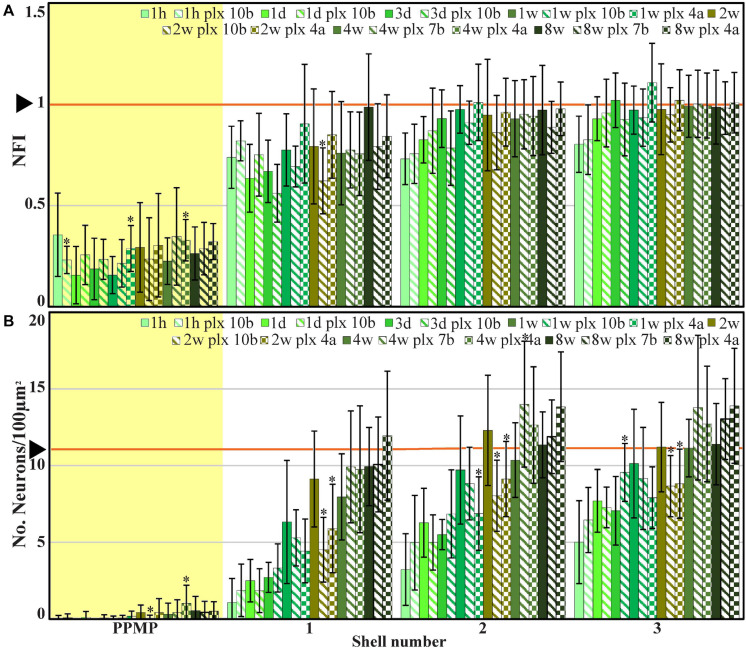
The average normalized fluorescent intensity (NFI) of neuronal cell bodies and neurites **(A)** and cell bodies alone (**B**, number of cell bodies/100 μm^2^) within and in contact with the perforated segment of the implanted PPMPs and in the four shells around it at different time points post-PPMP implantation. Homogeneous columns depict control cortices; diagonal stripes depict cortices from rats fed with PLX5622 7–10 days before PPMP implantation and thereafter; checkered columns depict cortices of rats fed with PLX5622 3–4 days after PPMP implantations and thereafter. The time post-platform implantation (1 h, 1 and 3 days, and 1, 2, 4, and 8 weeks) is coded by the darkening of the column colors as indicated by the legend on top of the histograms. The distance of the measured averages from the MEA platform is given by the shell number. Each shell is 25 μm wide (as illustrated in Figure 1). Vertical lines correspond to one standard deviation. The orange line pointed by an arrow head depicts the normal NFI value of the control neurons **(A)** and the average number of neuronal cell bodies/100 μm^2^ in the control cortices **(B)**. Asterisks indicate significant differences of *P* < 0.01 between the value of PLX5622-treated rats and controls at the same point in time. Similar data for the tip segment of the PPMP are shown in Supplementary Figure 1. For complete neuronal NFI density maps of the nine shells around the implant, see Supplementary Figure 2.

To that end, we examined immunohistological sections (from which the data on microglia and astrocytes were analyzed) in which neurons were co-labeled by NeuN and NF (which labels the cell bodies and neurites, respectively) at different points in time post-PPMP implantation (Figures 8, 9). The neuronal distribution was analyzed in two ways. First, the spatiotemporal distribution of the neuronal cell bodies along with their neurites (axons and dendrites) were mapped and analyzed by the NFI protocols, and then the neuronal cell bodies alone were counted by identifying the characteristic DAPI labeling of the neuronal nuclei heterochromatin, the nuclei shapes and sizes, along with NeuN labeling, as described previously by our laboratory (Huang et al., 2020).

The results depicted in Figure 9A indicate that the NFI values and spatiotemporal distributions of rats fed with PLX5622 chow for 7–10 days before PPMP implantation and onward, or 3–4 days after it and onward, did not differ significantly from the control rats. For example, 1 h after PPMP implantation and thereafter, the neuronal NFI intensity within the perforated platforms remained in the range of 0.16 ± 0.14 to 0.36 ± 0.21 (Figure 9A). These NFI levels mainly reflected the auto-fluorescence of the polyimide platform and the contribution of the adhering neurites. Interestingly, during the first 3 days post-PPMP implantation, the NFI values in the first shell (0–25 μm from the electrode’s surface) showed a trend toward reduction in both the control and PLX5622 pre-fed rats (Figure 9A). This might reflect necrotic processes and the removal of neuronal debris. Recall that alongside the reduced neuronal NFI in the first, second, and third shells around the implant, the microglia density in the first shell declined significantly 3 days post-implantation, from 16.6 ± 3.1 to 1.3 ± 1 cells/100 μm^2^ in the PLX5622 pre-fed rats. Thus, the observed reduction in neuronal NFI occurred prior to the appearance of the PLX-insensitive microglia cloud. This observation suggests that removal of neuronal debris by microglia is not critical. Thereafter, from 1 week post-PPMP implantation and onward, the neuronal NFI values gradually increased in shells 1, 2, 3, and 4 (0–100 μm) in both the control and PLX5622 pre-fed rats. This gradual NFI recovery indicates that neuro-regenerative processes took place probably in the form of neurite outgrowths (see next paragraph) in the vicinity of the implant. It should be noted that the neuronal NFI values of rats fed with PLX5622 4 days after PPMP implantation had similar NFI values on the seventh-day post-implantation and onward to the control. Overall, the neuronal NFI recovered to control values from shell 3 (50–75 μm) and onward whereas the neuronal NFI values in shells 1 and 2 recovered to 76–99% of the controls within 4–8 weeks after implantation in both the control and the PLX5622-fed rats.

Recall that the analyzed NFI values integrated the fluorescence signals generated by the neurites and the cell bodies. It is therefore reasonable to assume that a large fraction of the recorded NFI values represented neurite regrowth.

To examine whether the density of the neuronal cell bodies also changed, we analyzed the distribution densities of the neuronal cell bodies as a function of distance and time after implantation in the control and the PLX5622-treated rats. This was done by counting the neuronal cell bodies within the PPMP and in shells 1, 2, and 3 and calculating their corresponding densities per 100 μm^2^. We found that in both the control rats and the rats fed with PLX5622 chow, a gradual recovery in the density of neuronal cell bodies was recorded. Interestingly, in contrast to the NFI values which appeared to decrease during the first 3 days after PPMP implantation, the number of cell bodies in shell 1 started to increase from the first day after implantation. In all other shells, the number of cell bodies gradually recovered to control by week 8 after implantation (Figures 3, 9).

It should be emphasized that the immunohistological identification of neurons in the vicinity of the platform’s surface (Figure 8) does not imply that the labeled cells maintained their excitable membrane properties or synaptic connectivity. Concerns have recently been raised as to the decline in excitable membrane properties and synaptic connectivity by released proinflammatory cytokines from activated microglia and astrocytes induced by implanted MEA platforms (Goss-Varley et al., 2017; Salatino et al., 2017b, 2019; Thompson et al., 2020).

Taken together, these observations suggest that the drop in the overall microglia density by PLX5622 and the PLX5622-insensitive microglia did not change the overall regenerative trends or densities of the neurons induced by PPMP implantation.

### Spontaneous Field Potential Activity Recorded From PLX5622-Treated Rats

**FIGURE 10:**
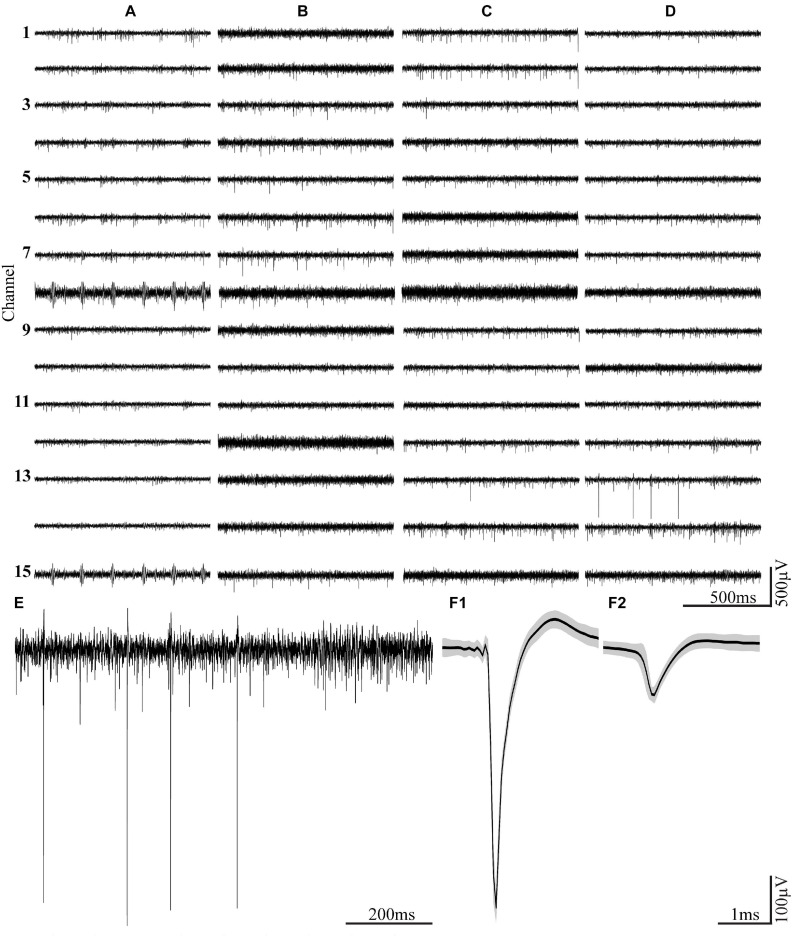
Examples of spontaneous FP recordings from a freely moving rat fed continuously with PLX5622 chow for a period of 16 weeks. Column **(A)** recordings of spontaneous FPs 1 h post-PPMP implantation, **(B)** 3 weeks post-implantation, **(C)** 9 weeks post-implantation, and **(D)** 16 weeks post-implantation. **(E)** An enlarged recording sweep at week 16. **(F1,F2)** Examples of average-sorted FP (black line) sweeps and one standard deviation (gray background) from the sample sweep, shown in panel **(E)**.

Earlier behavioral studies using mice and our own observations have demonstrated that PLX5622 feeding for up to 4 months does not lead to deterioration in the basic behavior of rats. This however does not imply that the excitable membrane properties and synaptic connectivity of neurons residing within the recording range of implanted electrodes (0–150 μm) are not altered (Goss-Varley et al., 2017; Salatino et al., 2017b, 2019; Thompson et al., 2020). To assess this issue, we next examined whether spontaneous FPs could be recorded from PLX5622-fed rats and compared their features to the controls. Recordings were made during the PPMP insertion, 1 h post-insertion, 2 and 4 days later, and thereafter weekly from awake, freely moving rats. Recordings continued for 8 weeks (and in two cases for up to 16 weeks) from control rats and rats continuously fed with PLX5622 chow (Figure 10). As reported by our laboratory (Huang et al., 2020), when using the PPMPs (under control conditions), the number of operating platforms, viable electrodes/PPMP, and recorded FP amplitudes deteriorated with time after implantation. Comparing these parameters among control group (*n* = 26 rats) and PLX5622 pre- and post-fed rats (*n* = 8 and 14 rats, respectively) did not reveal significant differences. The average noise level of the implanted PPMPs was 19.7 ± 3.09 μV (276 channels), and the signal-to-noise ratio ranged from 5 to 16. A comparison of the average spontaneous FP amplitudes recorded from the control and PLX5622-treated rats 1 h after PPMP implantation showed that they were similar [176 ± 52 μV (40 rats, 466 channels) in control rats and 171 ± 42 μV (8 rats, 94 channels) in PLX5622 pre-fed rats]. This suggests that the elimination of cortical microglia prior to PPMP implantation did not significantly damage the excitable membrane properties of the neurons. The overall average spontaneous FP amplitudes in the control and PLX5622-fed rats over a period of 8 weeks were also similar [in control rats 185 ± 55 μV (26 rats, 613 channels), in PLX5622 pre-fed rats 186 ± 36 μV (8 rats, 275 channels), and in rats post-fed PLX5622 160 ± 58 μV (13 rats, 270 channels)].

Importantly, since the recorded FPs in PLX5622-fed rats were spontaneously generated, it is conceivable that neurons in the vicinity of the implant were synaptically linked to other neurons. Note that FP recordings are not sufficient to reveal biophysical details. Nonetheless, these data imply that the PLX5622 treatment and the ensuing immunohistological alteration were not associated with greater damage than that inflicted by control PPMP implantations.

## Discussion

It is generally believed that mitigating the neuroinflammatory FBR induced by MEA-platform implants is required to improve the durability and recording/stimulation qualities of FPs for basic brain research and clinical applications. Nonetheless, despite extensive efforts, the relationships between the dimensions, immunohistological and biophysical characteristics of the FBR and FP recording qualities, and yield remained obscure (Kozai et al., 2014b, 2015b; McCreery et al., 2016; Du et al., 2017; Salatino et al., 2017a; Michelson et al., 2018).

Microglia, the resident macrophage of the CNS, serve as the first line of CNS defense against invading pathogens, sterile TBI, SCI, and injury caused by MEA implants. As microglia activation temporally precedes the activation of astrocytes (Polikov et al., 2005; Huang et al., 2020), actively migrating to the borders of the injury site or the MEA implant (Polikov et al., 2005; Kozai et al., 2012b; Potter et al., 2013; Campbell and Wu, 2018; Huang et al., 2020) and communicating with astrocytes and neurons (Biran et al., 2005; Block et al., 2007; Ereifej et al., 2018), they are perceived as downstream elements in the response to TBI, SCI, and MEA platform implants.

By combining the use of the brain permeant CSF1R inhibitor PLX5622 (Spangenberg et al., 2019) which eliminated approximately 89–94% of the female Sprague Dawley rats’ cortical microglia and a relatively large footprint PPMP that could be thin-sectioned along with the surrounding tissue (Huang et al., 2020), we examined here from a basic-research perspective the prevailing view that microglia serve key roles in sensing and translating the local tissue damage generated by MEA implants in what appears to be an orchestrated inflammatory FBR cascade and evaluated their role in the deterioration of FP recordings.

The main conclusions are as follows: (I) inflammatory FBR to PPMP cortical implantation is formed by astrocytes in microglia-depleted cortices. The time course and extent of astrocyte encapsulating scar around the implant in a microglia- depleted environment was indistinguishable from the control experiments (that is, in the presence of microglia). This observation raises the possibility that the roles of microglia in the FBR to PPMP implantation are redundant. (II) The rate and extent of neuronal degeneration (in association with the PPMP implantation) and the ensuing recovery of both the neuronal cell bodies and their neurites progress in PLX5622-fed rats and the control experiments in a similar manner. This suggests that microglia do not play critical functions in clearing away cellular debris or providing a critical protective neuronal environment. (III) Implantation of PPMP to control or to PLX5622-fed rats triggered a local surge of microglia mitosis on day 5 post-PPMP implantation within the pores of the platform in contact with its surface as well as within a 100-μm-thick shell around it. The daughter microglia were PLX5622 insensitive with an estimated short half life time of 1–2 weeks. Importantly, the density of microglia adhering to the PPMP (included in the first shell count) was significantly reduced with respect to the control rats. Nonetheless, the density of astrocytes adhering to the PPMP surfaces increased. This suggests that astrocyte branches that adhere to the PPMP surfaces replace the microglia insulation at about 2 weeks after PPMP implantation. (IV) Although substantial neuronal recovery (neurites and cell bodies) occurred in shells 1–6 (0–150 μm), this was not associated with increased FP amplitudes or yield. This observation is consistent with a model assuming that the attenuation of FP is generated by the seal resistance formed by a biofouling layer or by the narrow extracellular space between the adhering microglia and astrocytes and the PPMP platform rather than across the width of the scar tissue (Huang et al., 2020).

In the next paragraphs, we discuss potential mechanisms that could account for the observations and the implications of this study to mitigating the FBR.

### Astrocytes

Taken together, our observations suggest that the astrocyte-based inflammatory FBR was induced by MEA platform insertion in cortices depleted of 89–94% of the microglia despite the importance ascribed to the microglia as the first line of CNS defense and the importance of microglia/astrocytes crosstalk (Biran et al., 2005; Block et al., 2007; Zhang et al., 2010; Liu et al., 2011; Liddelow et al., 2017; Ereifej et al., 2018; Bellver-Landete et al., 2019; Jha et al., 2019). Although we cannot reject the possibility that the PLX5622-insensitive microglia cloud and the microglia attached to the PPMP released sufficient molecular signals to recruit astrocytes to the inflammatory processes (Hanisch and Kettenmann, 2007; Perry et al., 2010), it seems plausible that in the context of the inflammatory encapsulation of MEA implants, microglia are redundant and that the encapsulation of the implant can evolve by activated astrocytes alone (see Figures 4, 5, 7).

In the context of the FBR generated by MEA implants, it was shown that pattern recognition receptors (PRRs), such as Toll-like receptors (TLRs) which recognize DAMPs released by invading pathogens or from injured tissues induce inflammatory responses by activating the innate immune systems (Bedell et al., 2018; Hermann and Capadona, 2018). Thus, TLR and adaptor protein CD14 bind DAMPs released by MEA-platform implantation and activate microglia to initiate the inflammatory FBR cascade. As TLR are also expressed by astrocytes (Bsibsi et al., 2002; Kong and Le, 2011), it is conceivable that released DAMPs activate astrocytes directly. The relatively slow, day-long delay in astrocyte activation, with respect to the microglia in both the control experiments and the PLX5622 treated-rats may reflect innate metabolic features of the astrocytes.

It is important to note that, as recently suggested by our laboratory, the presence of adhering microglia (in control rats) and astrocytes (in control and PLX5622-treated rats) to the PPMP surfaces electrically insulate the electrodes from the surrounding neurons and thereby reduce the FP recording qualities (Huang et al., 2020).

### Neurons

The immunohistological results presented in this manuscript showed that in both control and PLX5622-treated rats 4–8 weeks post-PPMP implantation, the neuronal somata densities recovered to 72 ± 25–108 ± 38% of the controls in the first shell (0–25 μm) and to 94 ± 22–126 ± 34% more distal from it in shells 2 and 3 (≤75 away from the electrode surfaces). Since spontaneous FPs, with features similar to the control recordings (rise, decay time, duration, amplitude, and shape) were recorded in the control and PLX5622-treated rats by PPMPs implanted for over 8 weeks, it is conceivable that significant losses or alterations of membrane excitable properties and synaptic connectivity among the neuronal population surrounding the MEA implant did not take place. As the amplitude of a potential generated by a point current source passively declines in the brain’s extracellular space at an approximate rate of 1/*r*^*x*^ where *r* is the distance from the current source and *x* is in the range of 1< × <2 (Malaga et al., 2016; Michelson et al., 2018), the observed recovery of neuron density and location was theoretically expected to be associated with increased FP amplitudes and yield. Nonetheless, in both the control and the PLX5622 experiments, the average amplitude of the recorded FPs was in the same range. The apparent discrepancy between these results and prevailing theories can be explained by assuming that a high impedance biofouling layer formed over the electrode surface (Sommakia et al., 2009, 2014; Malaga et al., 2016) and/or that a seal resistance formed by the narrow extracellular space between the microglia and astrocytes that adhered to the electrodes partially or totally insulated the electrodes from the surrounding neurons (Huang et al., 2020).

### Microglia

Further to the extensive documentation of the cell biological mechanisms underlying microglia activation and migration toward a MEA platform implant (Kozai et al., 2012b, 2014a, 2016a, b; Eles et al., 2017, 2018; Wellman and Kozai, 2018), the present study emphasizes that the insertion of PPMP into the cortical parenchyma triggered a transient surge of local microglia mitosis in the PLX5622-treated (either pre- or post-fed PLX5622) and the control rats. This microglial-mitotic surge coincided with increased mitosis of astrocytes and oligodendrocytes but to a significantly lower extent (astrocytes 9–15% and oligodendrocytes 8% of the total dividing cells at this location and point in time). The daughter microglia differed from the “resting” microglia by being both PLX5622 insensitive and by having a significantly shorter *T*_1__/__2_. Whereas the *T*_1__/__2_ of resting microglia is 7.5–15 months (Fuger et al., 2017; Tay et al., 2017; Zhan et al., 2019), the half-life time of the daughter microglia that appears after electrode implantation is estimated to be in the range of 1–2 weeks. The observation that microglia mitosis is confined to the PPMP itself and to a ∼100-μm-thick shell around it suggests that diffusible signals released from injured and degenerating cells around the implant activated mitosis that generated the PLX5622-insensitive microglia cloud. With time, the microglia mitosis around the implant subsided and 2–8 weeks post-implantation the density of microglia in the first to fourth shells around it (0–100 μm) declined to pre-PPMP insertion levels; i.e., to 4.9 ± 1.1 microglia/100 μm^2^ in the control experiments and 2.3/1.1 ± 1.8/0.8 microglia/100 μm^2^ in the PLX5622 experiments (pre- and post-implantation PLX5622-fed rats, Figures 4, 5 and Supplementary Figures 1, 2). The increase and decline in microglia densities is consistent with the assumption that the release of DAMPs by damaged and degenerating cells triggers the mitotic surge (Venereau et al., 2015). Removal of the degenerating cells–possibly by astrocytes–through phagocytosis may eliminate the source of the mitotic signaling. The elimination of the mitotic factors together with the short *T*_1__/__2_ of the daughter PLX-insensitive microglia led to the relatively rapid decline in the microglia density around the implants.

It is interesting to note that while the microglia density in shells around the PPMP decreased consistently with the *T*_1__/__2_ from days 7–14, the microglia density within the PPMP remained high in both the control and the continuously fed rats with PLX5622 chow (425% in control, 446% pre-fed and 346% post-fed of the control microglia baseline densities, Figures 4, 5, even 8 weeks after implantation). A tentative mechanism to account for this observation could be related to the cell-biological effects of the platform’s stiffness relative to the surrounding cells on the microglia. Earlier studies have shown that the degree of substrate stiffness to which the microglia adhere leads to changes in the cell biological features (Moshayedi et al., 2014; Bollmann et al., 2015).

Of interest, the genuine source of the PLX5622-insensitive Iba-1/TMEM119 microglia cloud within the platform pores and around it is not known. Delayed migration of PLX5622-insensitive microglia from the parenchyma toward the implant is unlikely as we have not observed a “centripetal” microglia density gradient to support such a process. Infiltration of blood-borne immune cells through bridged BBB is a conceivable mechanism that should be further studied (Winslow and Tresco, 2010; Winslow et al., 2010; Saxena et al., 2013; Ravikumar et al., 2014; Bedell et al., 2018).

In context with the limitations in the electrophysiological use of MEA platforms attributed to the initiation and perpetuation of the inflammatory encapsulation cascade, the reported observations suggest that the FBR develop, at different rates, by parallel and independent activation of the microglia and the astrocytes. Whereas the experimental observations raise doubts as to the critical downstream roles of microglia in inflammatory scar formation, the seal resistance that obstructs electrophysiological recording is formed by microglia and astrocytic branches adhering to the PPMPs (Huang et al., 2020). Thus, to prevent the formation of seal resistance insulation would require concomitantly inhibiting the adhesion of both microglia and astrocytes to the implant surfaces. Only inhibiting the adhesion of the microglia or only the astrocytes might be insufficient to prevent the formation of seal-resistant electrical obstruction.

The reduction of microglia by PLX5622 proved to be an effective way to reduce microglia adhesion to the implant surfaces and thus could be used as one drug to target the microglia. Although systemic delivery of the drug in a feeding diet proved to be effective, its potential use needs to be enhanced by limiting its effects to the immediate vicinity of the implant by functionalizing the implant surfaces with the drug in a way that supports slow release for long periods of time.

Concomitant targeting of microglia and astrocytes could be achieved in a number of ways; for example, by inhibiting downstream DAMP receptors on both the microglia and astrocytes ahead of MEA implantation, as was recently examined by the Jeffrey R. Capadona laboratory (Bedell et al., 2018; Hermann and Capadona, 2018; Hermann et al., 2018) or by preventing the mitotic surge of microglia from occurring and the astrocytes from extending their branches or by concomitant inhibition of microglia and astrocyte adhesion to the electrode surfaces (Azemi et al., 2008, 2011; Eles et al., 2017). Note that a dual-targeted approach designed to interfere with the activation of microglia and astrocytes might lead to accumulation of cell debris along the path of the platform insertion and thereby impede regeneration processes.

Finally, it is crucial to recall that although it is conceivable that the effects of the PLX5622 diet on the inflammatory cascade induced by the PPMPs can be generalized, implants in different brain regions, made of different materials, size, shapes, and microarchitecture may elicit different cascades.

## Data Availability Statement

The original contributions presented in the study are included in the article/Supplementary Material, further inquiries can be directed to the corresponding author/s.

## Ethics Statement

The animal study was reviewed and approved by Committee for Animal Experimentation at the Institute of Life Sciences of the Hebrew University of Jerusalem.

## Author Contributions

NS headed the fabrication of the perforated polyimide MEA platforms. MJ and AS implanted the platforms. AS and HE conducted the electrophysiological recording sessions, prepared and analyzed the immunohistological sections and electrophysiological results. HE prepared all the figures. MS conceived, designed, and supervised the project, and wrote the manuscript. All authors contributed to the article and approved the submitted version.

## Conflict of Interest

The authors declare that the research was conducted in the absence of any commercial or financial relationships that could be construed as a potential conflict of interest.
