# Supplementary material for: Inflammatory Foreign Body Response Induced by Neuro-Implants in Rat Cortices Depleted of Resident Microglia by a CSF1R Inhibitor and Its Implications
Source: Front Neurosci. 2021 Mar 26;15:646914. doi: 10.3389/fnins.2021.646914 (PMC8032961; doi:10.3389/fnins.2021.646914)
Supplement: Supplementary file 1 [file Table_1.docx]

**
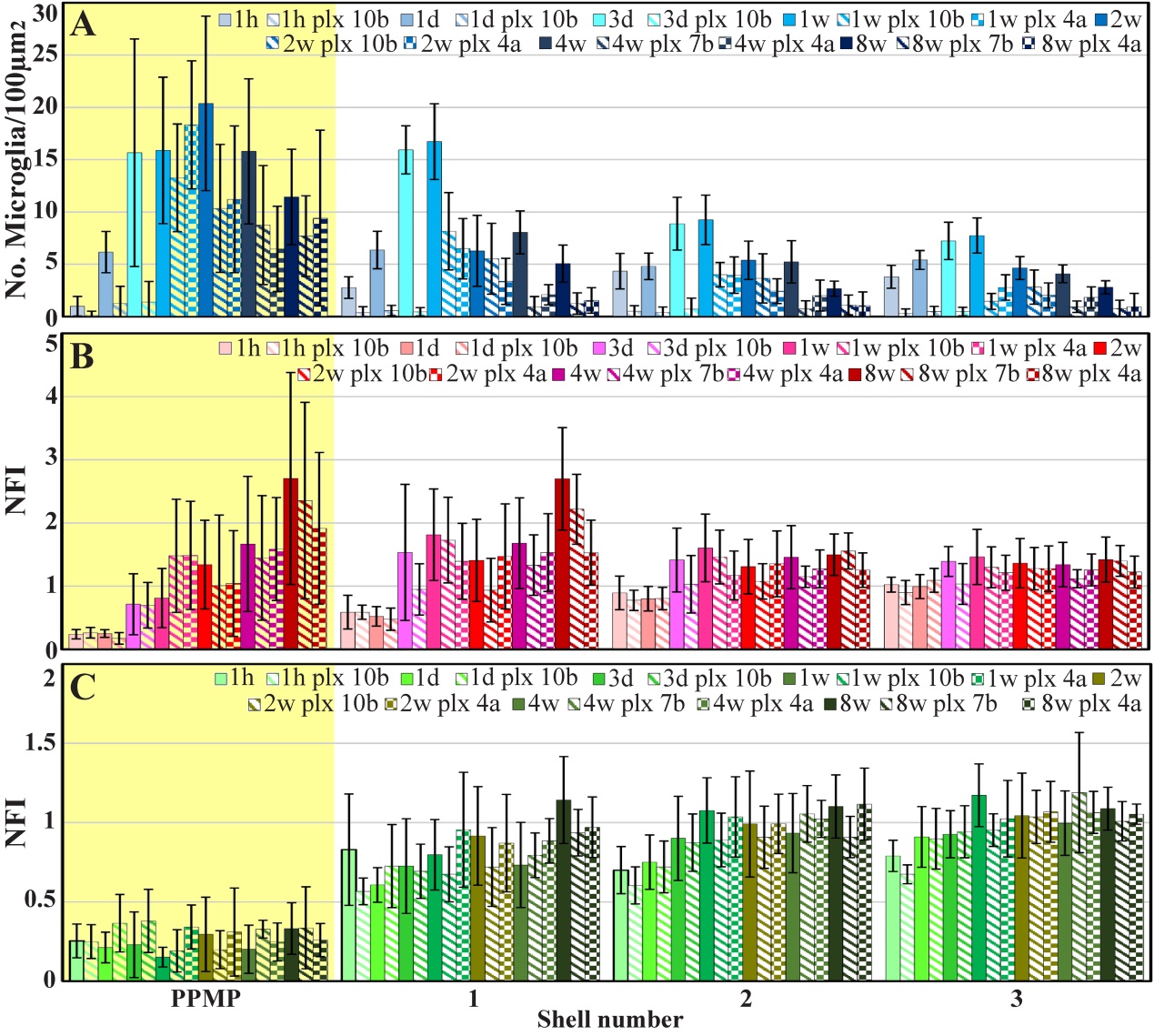
Supplementary figures:**

**Supplementary figure S1. Histograms depicting the average number of microglia per 100µm^2^ (A, blue), the average Normalized Fluorescent Intensity (NFI) of, astrocytes (B, red) and neurons (C, green) within and around the platform’s tip.** The time post platform implantation is coded by the darkening of the column color as indicated by the legend on top of each histogram. The average NFI values within the platforms (E) are emphasized by the yellow background. The distance of the average NFI from the MEA platform is given by shell number. Each shell is 25µm wide (as illustrated in Figure 1). Vertical lines correspond to one standard deviation.

**
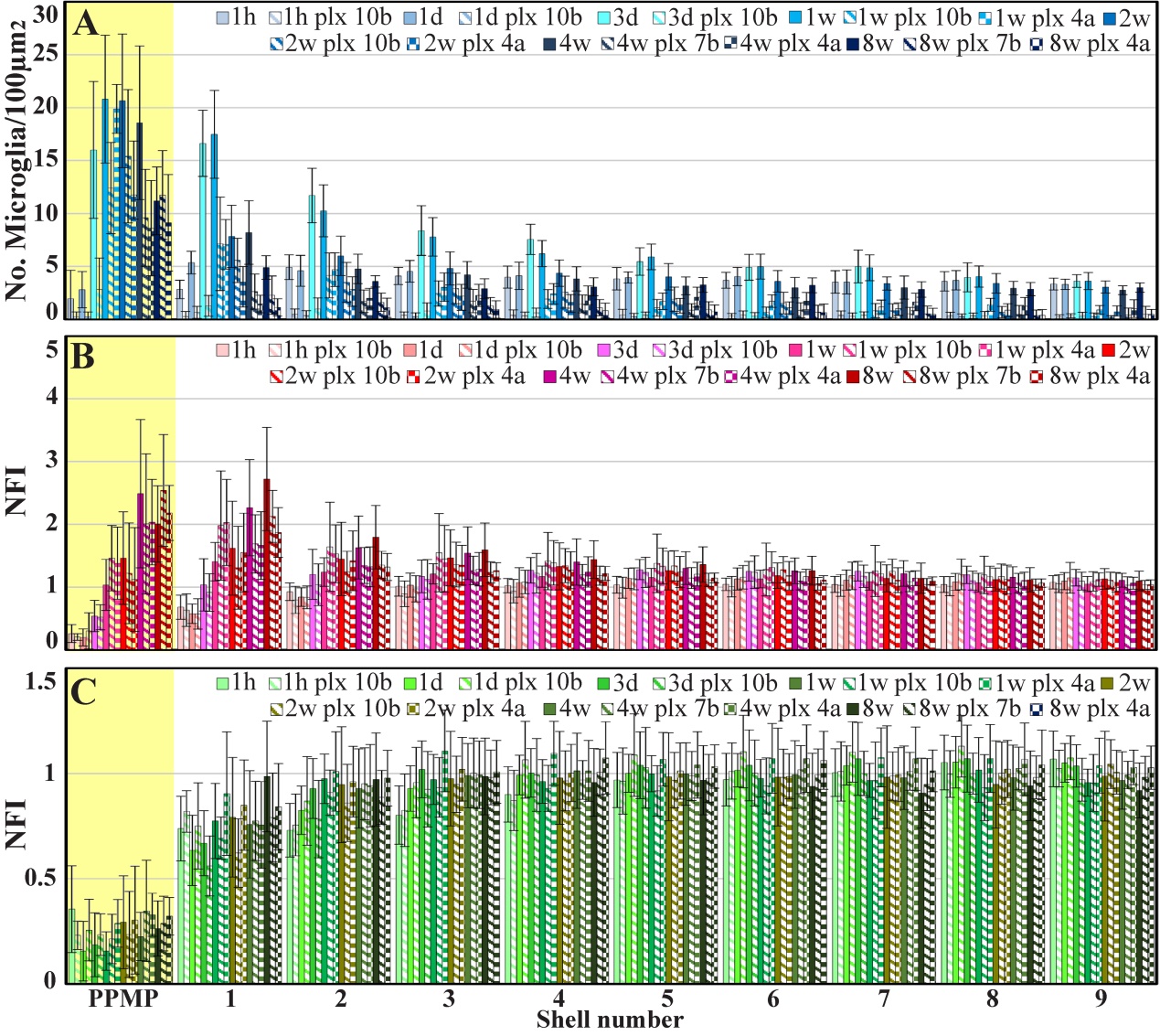
**

**Supplementary figure S2. Histograms depicting the average number of microglia per 100µm^2^ (A, blue), the Normalized Fluorescent Intensity (NFI) of astrocytes (B, red) and neurons (C, green) within and in 9 shells around the platform’s perforated segment.** The time post platform implantation is coded by the darkening of the column color as indicated by the legend on top of each histogram. The average NFI values within the platforms (E) are emphasized by the yellow background. The distance of the average NFI from the MEA platform is given by shell number. Each shell is 25µm wide (as illustrated in Figure 1). Vertical lines correspond to one standard deviation.

| **Time** | | **1 hour** | **1 day** | **3 days** | **1 week** | **2 weeks** | **4 weeks** | **8 weeks** |
| --- | --- | --- | --- | --- | --- | --- | --- | --- |
| **µG#** | Tip | 11/3 | 8/4 | 10/3 | 10/4 | 12/3 | 22/5 | 11/4 |
|  | Porous | 17/4 | 15/4 | 13/3 | 17/6 | 15/4 | 27/5 | 19/4 |
| **As** | Tip | 11/3 | 8/4 | 7/3 | 10/5 | 28 /9 | 20/5 | 11/4 |
|  | Porous | 19/4 | 18/4 | 10/2 | 18/7 | 29/10 | 26/5 | 18/4 |
| **Ne** | Tip | 11/3 | 7/3 | 10/3 | 6/3 | 24/7 | 19/5 | 10/4 |
|  | Porous | 21/4 | 18/4 | 12/3 | 16/6 | 24/7 | 22/ 5 | 18/4 |
| **Ne**# | Porous | 21/4 | 18/4 | 11/3 | 16/6 | 24/7 | 22/5 | 18/4 |

**Control**

**PLX 7-10 days before implantation**

| **Time** | | **1 hour** | **1 day** | **3 days** | **1 week** | **2 weeks** | **4 weeks** | **8 weeks** |
| --- | --- | --- | --- | --- | --- | --- | --- | --- |
| **µG#** | Tip | 10/4 | 14/4 | 9/3 | 10/5 | 28/7 | 20/4 | 14/3 |
|  | Porous | 18/5 | 16/4 | 11/3 | 14/5 | 24/7 | 23/4 | 25/4 |
| **As** | Tip | 12/4 | 13/4 | 7/2 | 8/4 | 24/7 | 8/4 | 9/3 |
|  | Porous | 20/5 | 13/4 | 10/3 | 11/5 | 19/7 | 16/4 | 20/4 |
| **Ne** | Tip | 10/3 | 13/4 | 9/3 | 8/4 | 23/7 | 7/3 | 9/3 |
|  | Porous | 10/4 | 13/4 | 12/3 | 11/4 | 24/7 | 12/4 | 16/4 |
| **Ne**# | Porous | 10/4 | 13/4 | 12/3 | 11/4 | 23/7 | 12/4 | 16/4 |

**PLX 3-4 days after implantation**

| **Time** | | **1 hour** | **1 day** | **3 days** | **1 week** | **2 weeks** | **4 weeks** | **8 weeks** |
| --- | --- | --- | --- | --- | --- | --- | --- | --- |
| **µG#** | Tip |  |  |  | 15/4 | 28/8 | 17/4 | 15/3 |
|  | Porous |  |  |  | 16/4 | 24/7 | 22/4 | 24/4 |
| **As** | Tip |  |  |  | 13/4 | 25/8 | 12/4 | 9/3 |
|  | Porous |  |  |  | 12/4 | 31/8 | 16/4 | 16/4 |
| **Ne** | Tip |  |  |  | 17/4 | 25/7 | 9/3 | 8/3 |
|  | Porous |  |  |  | 17/4 | 26/7 | 22/4 | 16/4 |
| **Ne**# | Porous |  |  |  | 17/4 | 24/7 | 22/4 | 16/4 |

**Supplementary Table S1.** Number of examined brain Slices (n) / Hemispheres (N). Ten optical sections were made per a single brain slice. µG - Microglia; As - Astrocytes and Ne- Neurons. # indicate hemispheres used for cell body counts, unmarked relate to hemispheres used for NFI values.

|  | 1 hour | 1 day | 3 days | 1 week | 2 weeks | 4 weeks | 8 weeks |
| --- | --- | --- | --- | --- | --- | --- | --- |
| Microglia# Porous- Shells E | **Control**  **1.97±2.67**  **PLX 10B**  ***0.25±0.46** | **Control 2.81±1.72**  **PLX 10B**  ***0.24±0.47** | **Control**  **16.01­­­±6.46**  **PLX 10B**  ***2.81±2.99** | **Control 20.80±6.04**  **PLX 10B**  ***12.41±4.3**  **PLX 4A**  **19.89±2.3** | **Control 20.64±6.31**  **PLX 10B**  ***15.40±6.3**  **PLX 4A**  ***11.76±5.08** | **Control 18.57±7.25**  **PLX 10B**  ***9.59±4.78**  **PLX 4A**  ***8.62±4.5** | **Control**  **11.19±3.2**  **PLX 10B**  **11.74±4.19**  **PLX 4A**  **9.12±4.56** |
| Microglia#  Porous- Shells 1 | **Control**  **2.83±0.87**  **PLX 10B**  ***0.29±0.46** | **Control 5.37±1.06**  **PLX 10B**  ***0.66±0.57** | **Control 16.63±3.12**  **PLX 10B**  ***1.28±0.98** | **Control 17.47±4.15**  **PLX 10B**  ***7.14±4.41**  **PLX 4A**  ***7.09±2.34** | **Control 7.84±2.93**  **PLX 10B**  ***5.57±2.09**  **PLX 4A**  ***3.51±1.48** | **Control**  **8.20±3**  **PLX 10B**  ***2.63±1.65**  **PLX 4A**  ***1.46±0.82** | **Control**  **4.90±1.12**  **PLX 10B**  ***2.28±1.81**  **PLX 4A**  ***1.13±0.82** |
| Astrocytes Porous - Shells E | **Control**  **0.26±0.14**  **PLX 10B**  **0.21±0.05** | **Control 0.2±0.14**  **PLX 10B**  **0.3±0.29** | **Control 0.54±0.25**  **PLX 10B**  **0.52±0.2** | **Control 1.04±0.41**  **PLX 10B**  **1.46±0.52**  **PLX 4A**  **1.38±0.58** | **Control 1.46±0.74**  **PLX 10B**  **1.22±0.8**  **PLX 4A**  **1.12±0.83** | **Control 2.49±1.18**  **PLX 10B**  **2.01±1.12**  **PLX 4A**  **2.04±0.68** | **Control 2.01±0.61**  **PLX 10B**  **2.54±0.89**  **PLX 4A**  **2.18±0.44** |
| Astrocytes Porous - Shells 1 | **Control**  **0.68±0.19**  **PLX 10B**  **0.63±0.26** | **Control 0.58±0.16**  **PLX 10B**  **0.57±0.24** | **Control 1.04±0.42**  **PLX 10B**  **0.92±0.3** | **Control 1.41±0.31**  **PLX 10B**  **1.98±0.87**  **PLX 4A**  ***2.03±0.69** | **Control 1.62±0.75**  **PLX 10B**  **1.31±0.66**  **PLX 4A**  **1.55±0.63** | **Control 2.26±0.77**  **PLX 10B**  ***1.69±0.46**  **PLX 4A**  ***1.66±0.54** | **Control 2.72±0.82**  **PLX 10B**  ***2.13±0.41**  **PLX 4A**  ***1.87±0.4** |
| Neurons Porous - Shells E | **Control 0.36±0.21**  **PLX 10B**  ***0.23±0.07** | **Control 0.16±0.14**  **PLX 10B**  **0.26±0.15** | **Control 0.19±0.15**  **PLX 10B**  **0.23±0.1** | **Control 0.16±0.09**  **PLX 10B**  **0.21±0.12**  **PLX 4A**  ***0.29±0.11** | **Control 0.29±0.22**  **PLX 10B**  **0.23±0.2**  **PLX 4A**  **0.3±0.26** | **Control 0.23±0.11**  **PLX 10B**  **0.35±0.24**  **PLX 4A**  ***0.33±0.1** | **Control 0.26±0.13**  **PLX 10B**  **0.29±0.13**  **PLX 4A**  **0.32±0.09** |
| Neurons Porous - Shells 1 | **Control 0.74±0.15**  **PLX 10B**  **0.82±0.1** | **Control 0.63±0.17**  **PLX 10B**  **0.75±0.2** | **Control 0.67±0.15**  **PLX 10B**  **0.56±0.14** | **Control 0.77±0.18**  **PLX 10B**  **0.69±0.1**  **PLX 4A**  **0.9±0.29** | **Control 0.79±0.28**  **PLX 10B**  ***0.62±0.16**  **PLX 4A**  **0.85±0.21** | **Control 0.76±0.26**  **PLX 10B**  **0.77±0.19**  **PLX 4A**  **0.76±0.21** | **Control 0.99±0.26**  **PLX 10B**  **0.79±0.21**  **PLX 4A**  **0.84±0.21** |
| Neurons# Porous - Shells E | **Control 0.04±0.19**  **PLX 10B**  **0.08±0.26** | **Control**  **0±0**  **PLX 10B**  **0.11±0.38** | **Control**  **0±0**  **PLX 10B**  **0.06±0.22** | **Control 0.04±0.16**  **PLX 10B**  **0.06±0.19**  **PLX 4A**  **0.18±0.34** | **Control 0.41±0.5**  **PLX 10B**  ***0.04±0.21**  **PLX 4A**  **0.43±0.9** | **Control 0.32±0.72**  **PLX 10B**  **0.41±0.84**  **PLX 4A**  ***1.03±1.17** | **Control 0.55±0.93**  **PLX 10B**  **0.43±0.72**  **PLX 4A**  **0.51±0.62** |
| Neurons# Porous - Shells E | **Control**  **1.08 ±1.56**  **PLX 10B**  **1.87±1.71** | **Control 2.5±1.38**  **PLX 10B**  **1.85±1.43** | **Control 2.72±0.98**  **PLX 10B**  **3.33±1.57** | **Control 6.33±4.01**  **PLX 10B**  **5.3±1.83**  **PLX 4A**  **4.44±2.08** | **Control 9.13±3.12**  **PLX 10B**  ***4.52±2.11**  **PLX 4A**  ***5.9±2.88** | **Control 7.96±2.81**  **PLX 10B**  **9.92±3.64**  **PLX 4A**  **9.76±4.13** | **Control 9.94±2.55**  **PLX 10B**  **10.08±3.09**  **PLX 4A**  **11.95±4.2** |

**Supplementary Table S2.** Mean ± one standard derivation of the average number of cells per 100µm^2^ (# microglia and neurons) or Normalized Fluorescent Intensity (NFI) (for astrocytes and neuron cell bodies and neurites) within the implanted platform (referred to as Shell E) and 0-25µm away from the platform’s surface (Shell 1). T-test was conducted for two-samples assuming unequal variances. P<0.01 Indicated by asterisks.

The average number of microglia per 100µm^2^ in control cortices was 2.63±0.34 (N=56)

The average number of neurons per 100µm^2^ was 11.6±1.79 (N=28)
